# Effects of Flavonoid Supplementation on Nanomaterial-Induced Toxicity: A Meta-Analysis of Preclinical Animal Studies

**DOI:** 10.3389/fnut.2022.929343

**Published:** 2022-06-14

**Authors:** Dongli Xie, Jianchen Hu, Tong Wu, Wei Xu, Qingyang Meng, Kangli Cao, Xiaogang Luo

**Affiliations:** ^1^College of Textile and Clothing Engineering, Soochow University, Suzhou, China; ^2^Shanghai Jing Rui Yang Industrial Co., Ltd, Shanghai, China; ^3^Shanghai Nutri-woods Bio-Technology Co., Ltd, Shanghai, China; ^4^Shanghai Pechoin Daily Chemical Co., Ltd, Shanghai, China; ^5^Shanghai Institute of Spacecraft Equipment, Shanghai, China

**Keywords:** nanomaterials, oxidative stress, inflammation, flavonoids, meta-analysis

## Abstract

**Background:**

Nanomaterials, widely applied in various fields, are reported to have toxic effects on human beings; thus, preventive or therapeutic measures are urgently needed. Given the anti-inflammatory and antioxidant activities, supplementation with flavonoids that are abundant in the human diet has been suggested as a potential strategy to protect against nanomaterial-induced toxicities. However, the beneficial effects of flavonoids remain inconclusive. In the present study, we performed a meta-analysis to comprehensively explore the roles and mechanisms of flavonoids for animals intoxicated with nanomaterials.

**Methods:**

A systematic literature search in PubMed, EMBASE, and Cochrane Library databases was performed up to April 2022. STATA 15.0 software was used for meta-analyses.

**Results:**

A total of 26 studies were identified. The results showed that flavonoid supplementation could significantly increase the levels of antioxidative enzymes (superoxide dismutase, catalase, glutathione, glutathione peroxidase, and glutathione-S-transferase), reduce the production of oxidative agents (malonaldehyde) and pro-inflammatory mediators (tumor necrosis factor-α, interleukin-6, IL-1β, C-reactive protein, immunoglobulin G, nitric oxide, vascular endothelial growth factor, and myeloperoxidase), and alleviate cell apoptosis (manifested by decreases in the mRNA expression levels of pro-apoptotic factors, such as caspase-3, Fas cell surface death receptor, and Bax, and increases in the mRNA expression levels of Bcl2), DNA damage (reductions in tail length and tail DNA%), and nanomaterial-induced injuries of the liver (reduced alanine aminotransferase and aspartate aminotransferase activities), kidney (reduced urea, blood urea nitrogen, creatinine, and uric acid concentration), testis (increased testosterone, sperm motility, 17β-hydroxysteroid dehydrogenase type, and reduced sperm abnormalities), and brain (enhanced acetylcholinesterase activities). Most of the results were not changed by subgroup analyses.

**Conclusion:**

Our findings suggest that appropriate supplementation of flavonoids may be effective to prevent the occupational detriments resulting from nanomaterial exposure.

## Introduction

The rapid advancement of nanotechnology has promoted the wide application of nanomaterials in many fields, such as the manufacturing industry, biomedicine, agriculture, water treatment, food additive, and cosmetics (1–5). The increased use makes nanomaterials unavoidably enter human bodies *via* various routes (including inhalation, skin absorption, ingestion, or injection) and then cause toxicities to different organs (e.g., lung, liver, kidney, brain, prostate, and testis) (6, 7). Therefore, it is urgently imperative to develop strategies to prevent and treat harmful effects of nanomaterials on human health.

Since pharmacological interventions are always prone to induce adverse side effects, nutrition modification has recently gained more attention as an important method for the prevention and treatment of diseases. Flavonoids that include six main subclasses [flavanones, flavones, flavonols, flavanols (monomer flavan-3-ols and polymer proanthocyanidins), anthocyanidins, and isoflavones] based on their molecular structures belong to a group of polyphenolic secondary metabolites in the plants (8, 9). Flavonoids can be ingested into the human diet by consuming fruits, vegetables, seeds, bark, roots, stems, leaves, flowers, and beverages (e.g., fruit juice, wine, beer, tea, coffee, and chocolate) (8, 9). Increasing evidence supports that flavonoid intake prevents the development and progression of diseases by exerting antioxidant and anti-inflammatory activities (10–12). It is also well known that activations of oxidative stress and inflammation are crucial mechanisms associated with tissue injuries induced by nanomaterials (13, 14). Therefore, daily dietary supplementation of flavonoids may represent a potential approach for the prevention and treatment of nanomaterial-induced toxicities; this hypothesis had been demonstrated by numerous animal experiments (15–18). However, the negative effects of flavonoids were also reported in some preclinical animal studies: Ali et al. found that, compared with rats that only received nickel oxide nanoparticles (NiONPs), the levels of hepatic oxidant malonaldehyde (MDA), antioxidant glutathione (GSH), and renal superoxide dismutase (SOD) were not significantly changed in rats undergoing a combined administration of flavones (apigenin) for 7 days, which was accompanied by no effects on liver (aspartate aminotransferase, AST; alanine aminotransferase, ALT) and renal (creatinine, urea, and blood urea nitrogen) functional biomarkers (19). Shahin et al. observed that the co-administration of flavonols (morin) for 1 or 2 weeks could not significantly alleviate titanium dioxide nanoparticles (TIO_2_NPs)-induced lipid peroxidation (MDA) in the testicular tissues and sperm abnormalities (20). The study of Dora et al. showed that, compared with iron oxide nanoparticle (IONP)-exposed rats, the levels of GSH and serotonin were not significantly increased in the brain homogenates of rats that were orally supplemented with flavonols (quercetin) at the dosages of 25 and 50 mg/kg. The oral additional administration of flavonols (quercetin) was also proved not to significantly reduce the levels of serum cytokine interleukin-6 (IL-6) relative to the zinc oxide nanoparticle (ZnONP)-intoxicated group (21). Abdelkarem et al. even found that the level of pro-inflammatory mediator tumor necrosis factor (TNF-α) was increased in the serum of the ZnONP-exposed rats after treatment with flavonols (quercetin) (22). Therefore, whether flavonoid supplementation exerts a protective role against nanomaterial-induced toxicities remains inconclusive.

In the present study, we performed a meta-analysis of all the available published preclinical studies to comprehensively explore the effects of flavonoid supplementation on oxidant stress-, inflammation-, and organ function-related indicators. Our study may provide a theoretical basis for clinical recommendation of flavonoid supplementation, especially for workers with occupational exposure to nanomaterials.

## Materials and Methods

### Literature Retrieval

This meta-analysis followed the guidelines of the preferred reporting items for systematic reviews and meta-analysis (PRISMA). Three databases, including PubMed, EMBASE, and Cochrane Library, were searched to compile relevant studies published up to April 2022. No limits were set on language. The search terms were designed by referring to the previous reports on flavonoids (23–25), including (“nanoparticle” OR “carbon nanotube” OR “graphene”) AND (“flavonoids” OR “flavonols” OR “flavanols” OR “flavan-3-ols” OR “flavanones” OR “flavones” OR “isoflavones” OR “isoflavanones” OR “anthocyanins” OR “proanthocyanidins” OR “anthocyanidin” OR “polyphenol” OR “cyanidin” OR “delphinidin” OR “malvidin” OR “pelargonidin” OR “catechins” OR “epicatechin” OR “epigallocatechin” OR “gallocatechin” OR “theaflavins” OR “thearubigins” OR “quercetin” OR “kaempferol” OR “myricetin” OR “myricitrin” OR “isorhamnetin” OR “galangin” OR “morin” OR “fisetin” OR “luteolin” OR “apigenin” OR “hesperetin” OR “naringenin” OR “naringin” OR “hesperidin” OR “isoflavonoid” OR “genistein” OR “daidzein” OR “baicalin” OR “silibinin” OR “taxifolin” OR “silymarin”) AND (animal). Additionally, the reference lists of the included studies and reviews were scanned to retrieve potential eligible studies.

### Inclusion and Exclusion Criteria

The participants, interventions, comparisons, outcomes, and study design (PICOS) criteria were used to include the eligible studies: (1) participants (P): murine; (2) intervention (I): the experimental group was administrated with nanomaterials and any kind of flavonoids (the dosage of which should be clearly reported); (3) comparison (C): the control group was only given nanomaterials; (4) outcomes (O): oxidative stress-related (MDA, SOD, and GSH; glutathione peroxidase, GPx; catalase, CAT; glutathione-S-transferase, GST; glutathione reductase, GR; nuclear factor erythroid 2-related factor 2, Nrf2), inflammation-related (TNF-α, IL-6, and IL-1β; C-reactive protein, CRP; immunoglobulin G, IgG; myeloperoxidase, MPO; nitric oxide, NO; vascular endothelial growth factor, VEGF), global health issues-related (body weight), organ injury-related (liver function: AST and ALT; ALP, alkaline phosphatase; renal function: albumin, urea, creatinine, uric acid, blood urea nitrogen; testis function: sperm count, sperm motility, sperm abnormalities, live sperm, and testosterone; luteinizing hormone, LH; follicle-stimulating hormone, FSH; 17β-hydroxysteroid dehydrogenase type, 17β-HSD; brain function: serotonin; acetylcholinesterase, AChE), apoptosis-related (TUNEL apoptotic index; mRNA expression levels of caspase-3; Fas cell surface death receptor, FAS; B-cell lymphoma-2, Bcl2; BCL2-associated X, apoptosis regulator, Bax), and DNA damage-related (tail length, tail DNA%) indicators; and (5) study design (S): controlled trials.

The exclusion criteria were as follows: (1) duplicate articles; (2) non-original research, such as case reports, reviews, editorials, letters, comments, and expert opinion papers; (3) *in vitro* and non-murine *in vivo* experiments; (4) interventions with plant extracts in which flavonoids were included, but detailed concentrations and dosages were unclear; (5) data could not be obtained or combined with other publications; and (6) irrelevant to the study topic. Two authors independently screened the literature, and any disagreements were resolved through discussion with a third reviewer.

### Data Extraction

Two reviewers independently extracted the following data from each eligible article: the first author, publication year, country of origin, animal species, sample size, type/dose of nanomaterials, type/dose/administration route/intervention duration of flavonoids, sample source for the analysis of outcomes, and data of outcomes of interest (mean ± standard deviation). The data presented in the bar graphs were estimated by using the digitizing software Engauge Digitizer 4.1^1^. Any discrepancies were resolved through discussion with a third reviewer.

### Quality Assessment

SYRCLE’s risk of bias tool was used to assess the quality of the included animal intervention studies (26). This tool consisted of 10 questions to reflect the source of bias from selection, performance, detection, attrition, reporting, and other aspects. The risk of bias was ranked as low, unclear, or high in each item when it was labeled as yes, no, or unknown to selected articles. The methodological quality was independently evaluated by two authors. Reviewers resolved discrepancies through discussion with a third reviewer.

### Statistical Analysis

STATA 15.0 software (STATA Corporation, College Station, TX, United States) was used for all statistical analyses. The effect size for all outcomes was expressed as standardized mean difference (SMD) and 95% confidence interval (CI). The heterogeneity between studies was assessed by using the Cochrane’s *Q*-square test and *I*^2^ statistic. A fixed-effects model was chosen to calculate the pooled results if obvious heterogeneity was present (*p* < 0.1 and *I*^2^ > 50%); otherwise, a random-effects model was selected. To explore the potential sources of between-study heterogeneity, subgroup analyses were performed based on nanomaterial types, subclasses, dosages, intervention durations, administration routes of flavonoids, sample sources, and animal species. Egger’s linear regression test was employed to investigate the publication bias. A trim-and-fill method was used to correct the pooled results in the presence of the publication bias (*p* < 0.05). The stability of the meta-analysis results was assessed by using a sensitivity analysis, which was based on the removal of one study at a time.

## Results

### Study Selection

**FIGURE 1:**
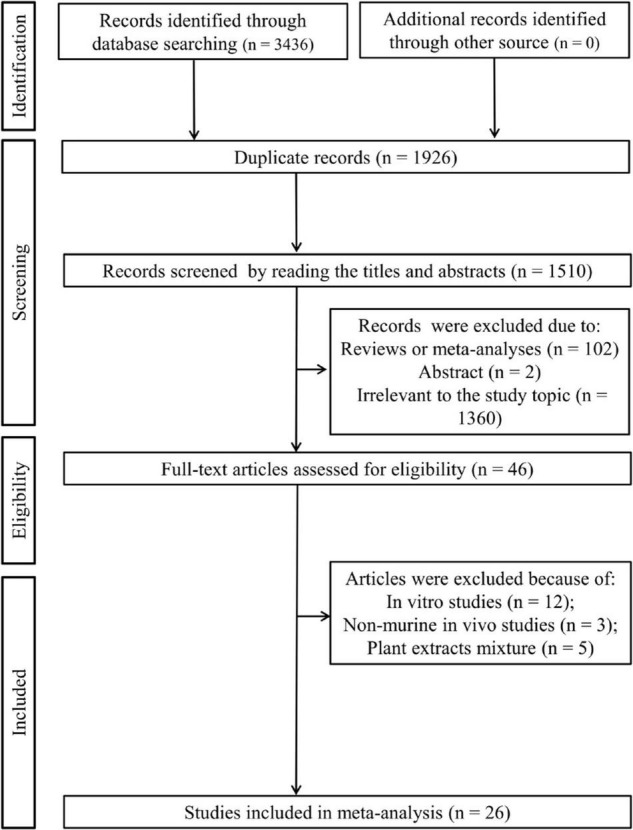
Flow chart of the literature search.

As shown in Figure 1, 3,436 articles were initially retrieved after a literature search in three electronic databases, of which 1,926 were duplicates. By browsing the titles and abstracts, 1,464 were excluded since 102 of them were reviews or meta-analyses, two of them were abstracts, and 1,360 studies were irrelevant to the study topic. The full text of the remaining 46 studies was downloaded and reviewed, after which 20 studies were removed because of the following reasons: *in vitro* studies (*n* = 12); non-murine *in vivo* experiments (*n* = 3); and plant extract mixture (*n* = 5). Finally, 26 studies were included in this meta-analysis (15–22, 27–44).

### Study Characteristics

The basic characteristics of the included articles are presented in Table 1. These 26 studies were published between 2012 and 2022. They were conducted in seven countries, including 12 in Egypt; six in Saudi Arabia; two in Iran, Nigeria, and China; and one in Korea and México. Twenty-two preclinical studies were carried out in rats, and the other four were conducted in mice. Three flavonoid subclasses were assessed in these studies, including flavonols (quercetin, *n* = 13; rutin, *n* = 2; morin, *n* = 3), flavanones (naringenin, *n* = 1; kolaviron, *n* = 2; hesperidin, *n* = 2; silibinin, *n* = 1), and flavones (apigenin, *n* = 3). The dosage of quercetin ranged from 5 to 200 mg/kg daily; two supplementation dosages were designed for rutin and kolaviron (50 and 100 mg/kg); three were designed for apigenin (20, 25, and 40 mg/kg); and only one dosage regimen was set for other flavonoid types (morin, 100 mg/kg; naringenin, 53 mg/kg; hesperidin, 100 mg/kg; silibinin, 20 mg/kg). Supplementation with flavonoids was used to protect the injuries induced by TiO_2_NPs (*n* = 7), ZnONPs (*n* = 6), silver nanoparticles (AgNPs, *n* = 3), carbon nanotubes (CNTs, *n* = 2), copper oxide nanoparticles (CuONPs, *n* = 1), gold nanoparticles (GNPs, *n* = 1), IONPs (*n* = 1), NiONPs (*n* = 1), silica dioxide nanoparticles (SiONPs, *n* = 2), and mesoporous silica nanoparticles (MSNPs, *n* = 1). Flavonoids were administered orally (*n* = 17), intraperitoneally (*n* = 5), or intragastrically (*n* = 4). The duration of intervention ranged from 1 to 12 weeks. The risk of bias was unclear or low for domains of all studies, indicating that the quality of included studies was acceptable (Table 2).

**TABLE 1:** Basic characteristics of the included articles.

Author	Year	Country	Animal species	No.	Nanomaterial type	Nanomaterial dose	Flavonoid type	Flavonoid subclass	Flavonoid dose	Flavonoid administration route	Flavonoid treatment duration	Outcomes
Elblehi et al. (42)	2022	Egypt	Rats	20	AgNPs	50 mg/kg	Quercetin	Flavonols	50 mg/kg	Orally	4.3 weeks	MDA, SOD, CAT, GSH, GPx, Nrf2, TNF-α, IL-6, Bax, Bcl2, AchE
Wang et al. (43)	2022	China	Mice	6	SiO_2_NPs	7 mg/kg	Apigenin	Flavones	20 mg/kg	Orally	2.4 weeks	MDA, SOD, Bax, Bcl2, Nrf2
El-Wafaey and Nafea (39)	2022	Egypt	Rats	16	ZnONPs	300 mg/kg	Naringenin	Flavanones	53 mg/kg	Orally	2 weeks	SOD, MDA, TNF-α, body weight, ALT, AST
Dora et al. (28)	2021	Egypt	Rats	16	IONPs	50 mg/kg	Quercetin	Flavonols	25 mg/kg	Orally	4.3 weeks	MDA, GSH, AchE, serotonin
Wang et al. (44)	2021	China	Mice	6	MSNPS	300 mg/kg	Apigenin	Flavones	40 mg/kg	Intraperitoneally	7 days	TNF-α, IL-6, creatinine, blood urea nitrogen
Ali et al. (19)	2021	Egypt	Rats	12	NiONPs	100 mg/kg	Apigenin	Flavones	25 mg/kg	Orally	1, 2, 4 weeks	MDA, GSH, SOD, AST, ALT, creatinine, blood urea nitrogen, urea
Abdelazeim et al. (27)	2020	Egypt	Rats	20	CuONPs	100 mg/kg	Quercetin	Flavonols	150 mg/kg	Intraperitoneally	3 weeks	ALT, AST, albumin, tail length, tail DNA%
Awogbindin (71)	2020	Nigeria	Rats	20	CNTs	1 mg/kg	Kolaviron	Flavanones	50, 100 mg/kg	Orally	2 weeks	MDA, SOD, CAT, GST, GPx, GSH, NO, MPO, TNF-α, ALT, AST, ALP, creatinine, urea
Adedara et al. (36)	2020	Nigeria	Rats	20	CNTs	1 mg/kg	Kolaviron	Flavanones	50, 100 mg/kg	Orally	2 weeks	MDA, SOD, CAT, GSH, GST, GPx, MPO, NO, TNF-α, AchE
Lim et al. (41)	2020	Korea	Mice	12	SiONPs	20 mg/kg	Silibinin	Flavanones	20 mg/kg	Orally	2 weeks	TNF-α, IL-6, IL-1β
Arisha et al. (37)	2019	Egypt	Rats	24	AgNPs	50 mg/kg	Morin	Flavonols	30 mg/kg	Orally	8 weeks	MDA, SOD, CAT, GPx, ALT, AST, ALP, creatinine, blood urea nitrogen, uric acid, sperm count, live sperm, sperm motility, sperm abnormalities, LH, FSH, testosterone, FAS, caspase-3, Bax, Bcl2
Hussein et al. (38)	2019	Egypt	Rats	20	TiO_2_NPs	300 mg/kg	Morin, rutin, morin + rutin	Flavonols	30 mg/kg, 100 mg/kg, 30 + 100 mg/kg	Intragastrically	4.3 weeks	MDA, SOD, CAT, GSH, sperm count, live sperm, sperm motility, sperm abnormalities, LH, FSH, testosterone
Abdelhalim et al., (29)	2018	Saudi Arabia	Rats	12	GNPs	50 μl	Quercetin	Flavonols	100 mg/kg	Intraperitoneally	1 week	MDA, GSH, creatinine, blood urea nitrogen, uric acid
Abdelhalim et al., (30)	2018	Saudi Arabia	Rats	10	GNPs	50 μl	Quercetin	Flavonols	200 mg/kg	Intraperitoneally	1 week	MDA, GSH, ALP, ALT
Alidadi et al. (32)	2018	Iran	Rats	16	TiO_2_NPs	50 mg/kg	Quercetin	Flavonols	75 mg/kg	Orally	3 weeks	MDA, SOD, CAT, body weight, creatinine, blood urea nitrogen, uric acid, TUNEL apoptotic index
Fadda et al. (16)	2018	Saudi Arabia	Rats	20	TiO_2_NPs	1000 mg/kg	Quercetin	Flavonols	200 mg/kg	Orally	3 weeks	MDA, TNF-α, IL-6, CRP, NO, IgG, VEGF, ALT, tail length, tail DNA%
Ansar et al. (18)	2018	Saudi Arabia	Rats	12	ZnONPs	600 mg/kg	Hesperidin	Flavanones	100 mg/kg	Orally	1 week	CAT, GPx, SOD, MDA, GSH
Shahin and Mohamed (20)	2017	Egypt	Rats	12	TiO_2_NPs	50 mg/kg	Morin	Flavonols	30 mg/kg	Intragastrically	1, 2, 3 weeks	MDA, GSH, TNF-α, body weight, testosterone, 17β-HSD, LH, FSH, FAS, Caspase 3, Bax, Bcl2
Ahmed and Hussein (40)	2017	Egypt	Rats	18	AgNPs	30 mg/kg	Rutin	Flavonols	50 mg/kg	Orally	8 weeks	MDA, GSH, CAT, SOD, GPx, serotonin
Khorsandi et al. (31)	2017	Iran	Mice	16	TiO_2_NPs	300 mg/kg	Quercetin	Flavonols	75 mg/kg	Intragastrically	6 weeks	MDA, SOD, CAT, body weight, testosterone, sperm count, sperm abnormality, TUNEL apoptotic index
Ansar et al. (17)	2017	Saudi Arabia	Rats	12	ZnONPs	600 mg/kg	Hesperidin	Flavanones	100 mg/kg	Orally	1 week	CAT, GPx, SOD, MDA, GSH, TNF-α, IL-6, IL-1β, CRP
Abdelkarem et al. (22)	2016	Egypt	Rats	20	ZnO NPs	600 mg/kg	Quercetin	Flavonols	200 mg/kg	Orally	3 weeks	NO, IgG, TNF-α, IL-6, CRP, tail length, tail DNA%
Hussein et al. (33)	2016	Egypt	Rats	20	ZnONPs	100, 400 mg/kg	Quercetin	Flavonols	100 mg/kg	Intragastrically	12 weeks	MDA, GSH, GPx, SOD, CAT, sperm count, live sperm, sperm motility, testosterone, 17β-HSD
González-Esquivel et al. (34)	2015	México	Rats	10	TiO_2_NPs	5 mg/kg	Quercetin	Flavonols	5 mg/kg	Intraperitoneally	2, 4 weeks	AST, ALT, MDA, Gpx, GR
Al-Rasheed et al. (15)	2013	Egypt	Rats	20	TiO_2_NPs	600, 1000 mg/kg	Quercetin	Flavonols	200 mg/kg	Orally	3 weeks	GSH, TNF-α, IL-6, CRP, NO, IgG, VEGF, creatinine, urea, uric acid
Faddah et al. (21)	2012	Saudi Arabia	Rats	20	ZnONPs	600, 1000 mg/kg	Quercetin	Flavonols	200 mg/kg	Orally	3 weeks	GSH, TNF-α, IL-6, CRP, NO, IgG, VEGF, body weight, creatinine, urea, uric acid

*TiO_2_NPs, titanium dioxide nanoparticles; CuONPs, copper oxide nanoparticles; IONPs, iron oxide nanoparticles; ZnONPs, zinc oxide nanoparticles; GNPs, gold nanoparticles; NiONPs, nickel oxide nanoparticles; AgNPs, silver nanoparticles; CNTs, carbon nanotubes; SiONPs, silica dioxide nanoparticles; MSNPs, mesoporous silica nanoparticles; MDA, malonaldehyde; SOD, superoxide dismutase; GSH, glutathione; GPx, glutathione peroxidase; CAT, catalase; GST, Glutathione-S-transferase; GR, glutathione reductase; Nrf2, nuclear factor erythroid 2-related factor 2; TNF, tumor necrosis factor; IL, interleukin; CRP, C-reactive protein; IgG, immunoglobulin G; MPO, myeloperoxidase; NO, nitric oxide; VEGF, vascular endothelial growth factor; ALT, alanine aminotransferase; AST, aspartate aminotransferase; ALP, alkaline phosphatase; LH, luteinizing hormone; FSH, follicle-stimulating hormone; 17β-HSD, 17β-hydroxysteroid dehydrogenase type; AChE, acetylcholinesterase.*

**TABLE 2:** Quality assessments based on SYRCLE’s risk of bias tool.

References	Selection bias			Performance bias		Detection bias		Attrition bias	Reporting bias	
	SG	BC	AC	RH	BI	ROA	BOA	IOD	SOR	Other
Elblehi et al. (42)	Unclear	Unclear	Unclear	Low	Unclear	Unclear	Unclear	Low	Low	Low
Wang et al. (43)	Unclear	Unclear	Unclear	Unclear	Unclear	Unclear	Unclear	Low	Low	Low
El-Wafaey and Nafea (39)	Unclear	Unclear	Unclear	Low	Unclear	Unclear	Unclear	Low	Low	Low
Wang et al. (44)	Unclear	Unclear	Unclear	Unclear	Unclear	Unclear	Low	Low	Low	Low
Dora et al. (28)	Unclear	Unclear	Unclear	Low	Unclear	Unclear	Unclear	Low	Low	Low
Ali et al. (19)	Unclear	Unclear	Unclear	Low	Unclear	Low	Unclear	Low	Low	Low
Abdelazeim et al. (27)	Unclear	Unclear	Unclear	Low	Unclear	Unclear	Unclear	Low	Low	Low
Awogbindin (71)	Unclear	Unclear	Unclear	Low	Unclear	Low	Unclear	Low	Low	Low
Adedara et al. (36)	Unclear	Unclear	Unclear	Low	Unclear	Low	Unclear	Low	Low	Low
Lim et al. (41)	Unclear	Unclear	Unclear	Unclear	Unclear	Unclear	Unclear	Low	Low	Low
Arisha et al. (37)	Unclear	Unclear	Unclear	Low	Unclear	Unclear	Unclear	Low	Low	Low
Hussein et al. (38)	Unclear	Low	Unclear	Low	Unclear	Unclear	Unclear	Low	Low	Low
Abdelhalim et al. (2018) (29)	Unclear	Unclear	Unclear	Unclear	Unclear	Unclear	Unclear	Low	Low	Low
Abdelhalim et al. (2018) (30)	Unclear	Unclear	Unclear	Unclear	Unclear	Unclear	Unclear	Low	Low	Low
Alidadi et al. (32)	Unclear	Low	Unclear	Low	Low	Unclear	Low	Low	Low	Low
Fadda et al. (16)	Unclear	Unclear	Unclear	Unclear	Unclear	Low	Unclear	Low	Low	Low
Ansar et al. (18)	Unclear	Low	Unclear	Low	Unclear	Unclear	Unclear	Low	Low	Low
Shahin and Mohamed (20)	Unclear	Unclear	Unclear	Low	Unclear	Unclear	Unclear	Low	Low	Low
Ahmed and Hussein (40)	Unclear	Unclear	Unclear	Low	Unclear	Unclear	Unclear	Low	Low	Low
Khorsandi et al. (31)	Unclear	Low	Unclear	Low	Low	Low	Low	Low	Low	Low
Ansar et al. (17)	Unclear	Unclear	Unclear	Unclear	Unclear	Unclear	Unclear	Low	Low	Low
Abdelkarem et al. (22)	Unclear	Unclear	Unclear	Low	Unclear	Unclear	Unclear	Low	Low	Low
Hussein et al. (33)	Unclear	Unclear	Unclear	Unclear	Unclear	Unclear	Unclear	Low	Low	Low
González-Esquivel et al. (34)	Unclear	Unclear	Unclear	Low	Unclear	Unclear	Unclear	Low	Low	Low
Al-Rasheed et al. (15)	Unclear	Unclear	Unclear	Low	Unclear	Unclear	Unclear	Low	Low	Low
Faddah et al. (21)	Unclear	Unclear	Unclear	Low	Unclear	Unclear	Unclear	Low	Low	Low

*SG, sequence generation; BC, baseline characteristics; AC, allocation concealment; RH, random housing; BI, blinding of investigators; ROA, random outcome assessment; BOA, blinding of outcome assessor; IOD, incomplete outcome data; SOR, selective outcome reporting.*

### Meta-Analysis

Since multiple dosages, intervention durations, and tissue samples were collected for some studies, the number of datasets in our meta-analysis was larger than the actual number of included studies. The detailed data that were extracted for each variable are summarized in Supplementary Table 1.

#### Effects of Flavonoid Supplementation on Oxidative Stress

**FIGURE 2:**
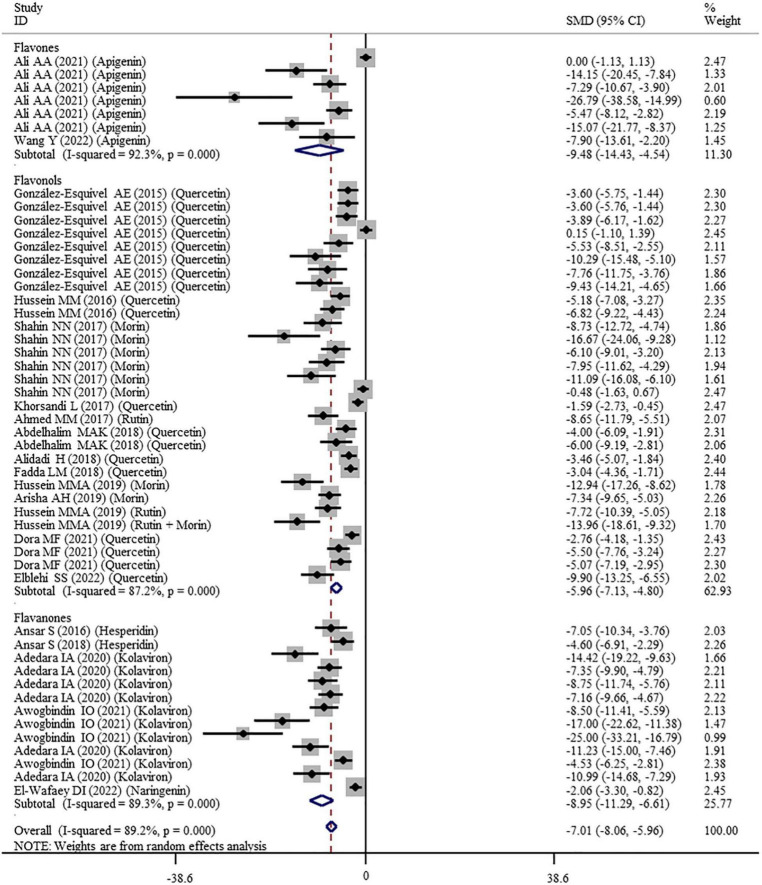
Forest plots to show the effects of flavonoid supplementation on MDA levels compared with the nanomaterial exposure group. MDA, malonaldehyde; SMD, standardized mean difference; CI, confidence interval.

**FIGURE 3:**
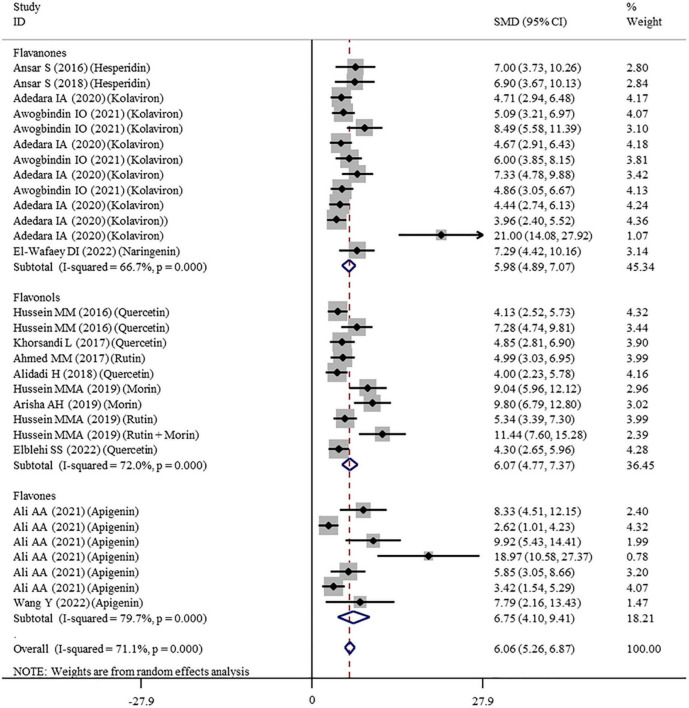
Forest plots to show the effects of flavonoid supplementation on SOD levels compared with the nanomaterial exposure group. SOD, superoxide dismutase; SMD, standardized mean difference; CI, confidence interval.

**FIGURE 4:**
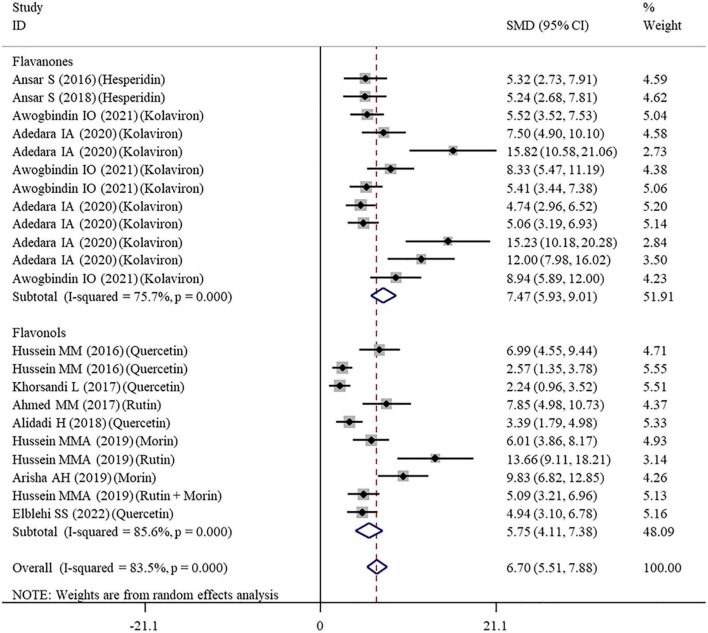
Forest plots to show the effects of flavonoid supplementation on CAT levels compared with the nanomaterial exposure group. CAT, catalase; SMD, standardized mean difference; CI, confidence interval.

The effects of flavonoid supplementation on MDA, SOD, CAT, GSH, GPx, GST, GR, and Nrf2 were reported in 50, 30, 22, 40, 25, 10, 9, and 2 datasets, respectively (Supplementary Table 1). The overall meta-analysis results showed that, compared with the nanomaterial-exposed group, nutritional interventions with flavonoids could significantly reduce the levels of pro-oxidant MDA (SMD = − 7.07; 95%CI, −8.06 to −5.96; *p* < 0.001; Figure 2) but increase the levels of antioxidant SOD (SMD = 6.06; 95%CI, 5.26 – 6.87; *p* < 0.001; Figure 3), CAT (SMD = 6.70; 95%CI, 5.52 – 7.88; *p* < 0.001; Figure 4), GSH (SMD = 5.64; 95%CI, 4.59 – 6.70; *p* < 0.001), GPx (SMD = 3.79; 95%CI, 2.57 – 5.00; *p* < 0.001), GST (SMD = 6.98; 95%CI, 5.37 – 8.60; *p* < 0.001), and Nrf2 (SMD = 7.27; 95%CI, 4.97 – 9.58; *p* < 0.001) (Table 3). The level of GR was not significantly different between two groups (*p* = 0.092) (Table 3).

**TABLE 3:** Meta-analysis results.

Variable	No.	SMD	95%CI	*P*_E_-value		*I* ^2^	*P*_H_-value	Model	Egger *p*
**Oxidative stress**									
MDA	50	–7.07	–8.06,–5.96	**<0.001**		89.2	<0.001	R	<0.001
SOD	30	6.06	5.26,6.87	**<0.001**	71.1	<0.001	R	<0.001	
CAT	22	6.70	5.52,7.88	**<0.001**	83.5	<0.001	R	<0.001	
GSH	40	5.64	4.59,6.70	**<0.001**	88.4	<0.001	R	<0.001	
GPx	25	3.79	2.57,5.00	**<0.001**	91.9	<0.001	R	0.001	
GST	10	6.98	5.37,8.60	**<0.001**	78.8	<0.001	R	<0.001	
GR	9	1.63	–0.26,3.52	0.092	88.0	<0.001	R	0.502	
Nrf2	2	7.27	4.97,9.58	**<0.001**	0.0	0.380	F	–	
**Inflammation**									
NO	16	–10.69	–12.88,–8.51	**<0.001**	86.7	<0.001	R	<0.001	
TNF-α	28	–6.61	–8.08,–5.15	**<0.001**	91.8	<0.001	R	<0.001	
IL-6	11	–4.10	–5.38,–2.82	**<0.001**	83.5	<0.001	R	<0.001	
IL-1β	3	–4.20	–5.45,–2.94	**< 0.001**		0.0	0.657	F	0.008
CRP	7	–5.24	–7.35,–3.14	**<0.001**	90.1	<0.001	R	<0.001	
IgG	6	–7.33	–9.81,–4.84	**<0.001**	84.4	<0.001	R	<0.001	
VEGF	5	–8.51	–11.50,–5.52	**<0.001**	82.6	<0.001	R	<0.001	
MPO	10	–13.58	–16.98,–10.18	**<0.001**	86.6	<0.001	R	<0.001	
**Apoptosis**									
TUNEL apoptotic index	2	–10.98	–17.63,–4.33	**0.001**	77.3	0.036	R	–	
FAS mRNA	7	–11.14	–14.01,–8.27	**<0.001**	59.5	0.022	R	0.010	
Caspase 3 mRNA	7	–10.46	–12.14,–8.79	**<0.001**	5.9	0.383	F	0.157	
Bax mRNA	12	–9.98	–11.73,–8.23	**<0.001**	54.9	0.011	R	<0.001	
Bcl2 mRNA	9	9.19	5.92,12.46	**<0.001**	88.7	<0.001	R	0.001	
**DNA damage**									
Tail length	3	–13.77	–16.45,–11.08	**<0.001**	34.4	0.218	F	<0.001	
Tail DNA%	3	–8.59	–10.31,–6.87	**<0.001**	25.4	0.262	F	0.003	
Body weight	8	1.11	0.26,1.97	**0.011**	76.5	<0.001	R	0.003	
**Liver function**									
ALT	14	–6.15	–8.77,–3.53	**<0.001**	94.8	<0.001	R	<0.001	
AST	12	–5.25	–7.44,–3.07	**<0.001**	92.5	<0.001	R	<0.001	
ALP	4	–11.26	–17.71,–4.80	**0.001**	94.3	<0.001	R	0.031	
Albumin	4	8.13	2.20,14.07	**0.007**	93.3	<0.001	R	0.008	
**Renal function**									
Urea	9	–3.56	–5.08,–2.04	**<0.001**	89.0	<0.001	R	<0.001	
Blood urea nitrogen	7	–5.76	–8.67,–2.84	**<0.001**	91.6	<0.001	R	0.001	
Creatinine	13	–5.22	–7.05,–3.38	**<0.001**	91.9	<0.001	R	<0.001	
Uric acid	7	–3.63	–5.68,–1.58	**0.001**	93.3	<0.001	R	<0.001	
**Testis function**									
Testosterone	10	6.97	4.48,9.46	**<0.001**	92.3	<0.001	R	<0.001	
FSH	7	2.61	–1.50,6.71	0.213	95.9	<0.001	R	0.784	
LH	7	3.09	–1.60,7.78	0.196	96.4	<0.001	R	0.239	
Sperm motility	6	7.64	4.06,11.22	**<0.001**	93.4	<0.001	R	<0.001	
Sperm count	7	5.95	2.93,8.97	**<0.001**	94.9	<0.001	R	0.001	
Sperm abnormalities	5	–7.73	–9.87,–5.60	**<0.001**	68.2	0.014	R	0.007	
Live sperm	6	7.05	1.32,12.78	**0.016**	96.9	<0.001	R	0.151	
17β-HSD	5	5.35	3.21,7.49	**<0.001**	84.6	<0.001	R	<0.001	
**Brain function**									
Serotonin	4	2.18	–0.57,4.93	0.120	91.0	<0.001	R	0.005	
AChE	10	4.38	0.49,8.28	0.027	96.5	<0.001	R	0.004	

*MDA, malonaldehyde; SOD, superoxide dismutase; GSH, glutathione; GPx, glutathione peroxidase; CAT, catalase; GST, Glutathione-S-transferase; GR, glutathione reductase; TNF, tumor necrosis factor; IL, interleukin; CRP, C-reactive protein; IgG, immunoglobulin G; MPO, myeloperoxidase; NO, nitric oxide; VEGF, vascular endothelial growth factor; Nrf2, nuclear factor erythroid 2-related factor 2; ALT, alanine aminotransferase; AST, aspartate aminotransferase; ALP, alkaline phosphatase; LH, luteinizing hormone; FSH, follicle-stimulating hormone; 17β-HSD, 17β-hydroxysteroid dehydrogenase type; AChE, acetylcholinesterase; SMD, standardized mean difference; CI, confidence interval; F, fixed-effects; R, random-effects; P_H_-value, significance for heterogeneity; P_E_-value, significance for treatment effects.*

*Bold values indicate the outcomes significantly changed by flavonoids.*

As shown in Table 3, a random-effects model was selected for the meta-analyses because there was evidence of heterogeneity in the analysis of all oxidative stress-related indicators. Therefore, subgroup analyses were performed for them. The pooled results showed that the significant beneficial effects of flavonoid supplementation on MDA (Figure 2), SOD (Figure 3), and CAT (Figure 4) were still found in all subgroups regardless of nanomaterial types, subclasses, dosages, intervention durations, administration routes of flavonoids, sample sources, and animal species (Supplementary Table 2). Although supplementation with all three flavonoid subclasses at various dosages and durations was found to reverse the decrease in GSH induced by nanomaterials (except for GNPs), the beneficial effects for brain, liver, kidney, and prostate tissues (except of testis) were mainly exerted by oral or intragastrical administration of quercetin, rutin, kolaviron, and apigenin but not morin (*p* = 0.377) and hesperidin (*p* = 0.176) (Supplementary Table 2). The significant effects on GPx of brain, liver, and testis tissues (not kidney) were only found in the subgroups with oral or intragastrical supplementation of flavanone (not flavonols) and flavonoid interventions for more than 2 weeks (Supplementary Table 2). Flavanone (kolaviron) supplementation was shown to significantly increase the levels of GST in brain, liver, and kidney tissues regardless of the used dosages (Supplementary Table 2). Flavanone (hesperidin) supplementation was found to increase the level of GR (SMD = 3.68; 95%CI, 1.71 – 5.65; *p* < 0.001) (Supplementary Table 2), but only one study reported this result, and thus, further confirmation was still needed.

#### Effects of Flavonoid Supplementation on Inflammation

**FIGURE 5:**
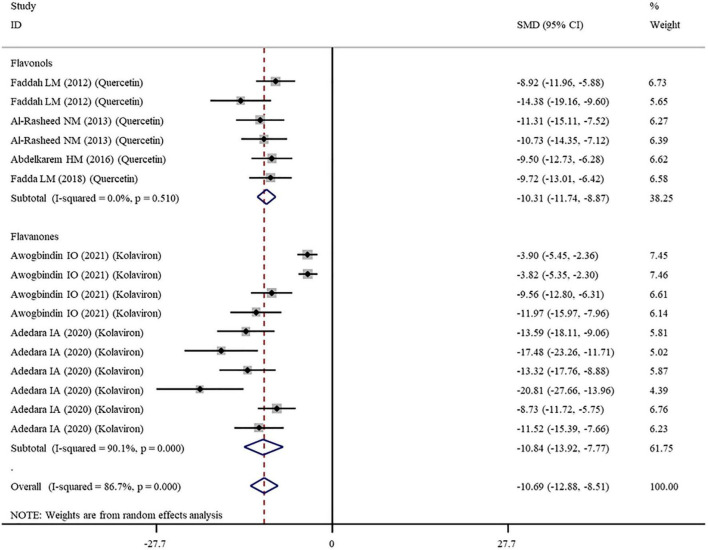
Forest plots to show the effects of flavonoid supplementation on NO levels compared with the nanomaterial exposure group. NO, nitric oxide; SMD, standardized mean difference; CI, confidence interval.

**FIGURE 6:**
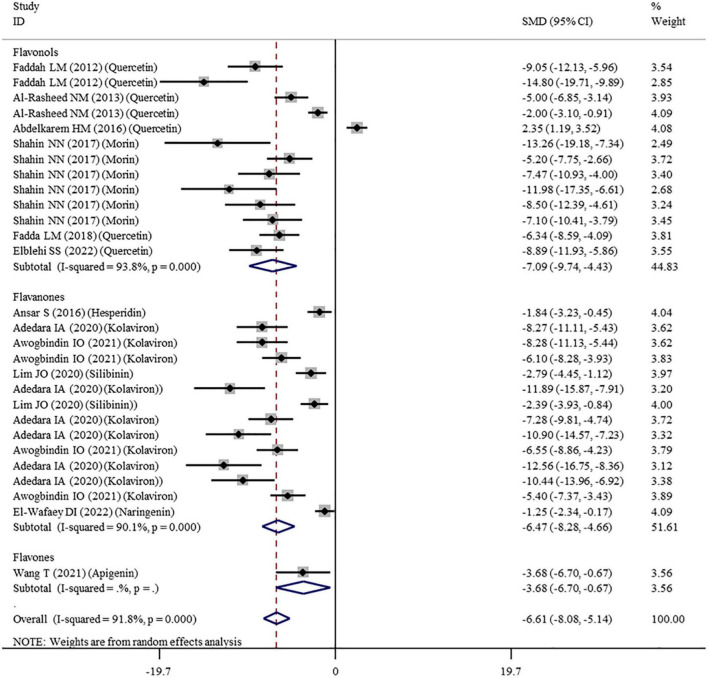
Forest plots to show the effects of flavonoid supplementation on TNF-α levels compared with the nanomaterial exposure group. TNF-α, tumor necrosis factor-α; SMD, standardized mean difference; CI, confidence interval.

**FIGURE 7:**
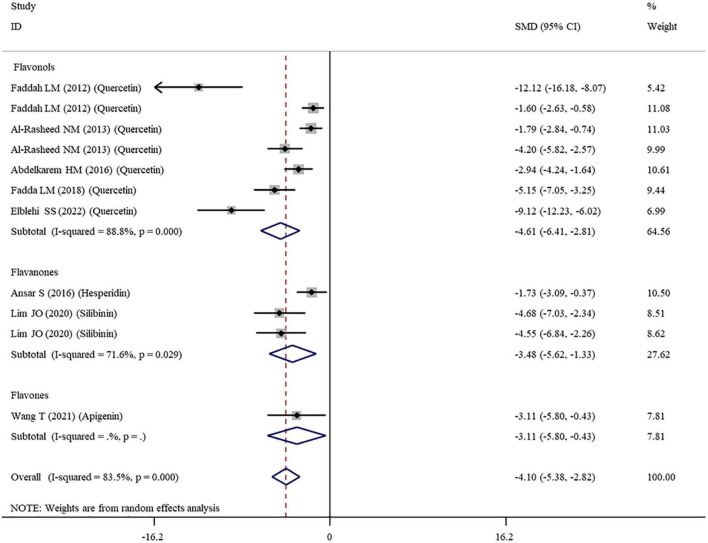
Forest plots to show the effects of flavonoid supplementation on IL-6 levels compared with the nanomaterial exposure group. IL, interleukin; SMD, standardized mean difference; CI, confidence interval.

The levels of NO, TNF-α, IL-6, IL-1β, CRP, IgG, VEGF, and MPO were measured in 16, 28, 11, 3, 7, 6, 5, and 10 datasets, respectively (Supplementary Table 1). The overall meta-analysis results indicated that co-administration of flavonoids to animals intoxicated with nanomaterials significantly ameliorated the elevation in the levels of pro-inflammatory NO (SMD = − 10.69; 95%CI, − 12.88 to − 8.51; *p* < 0.001; Figure 5), TNF-α (SMD = − 6.61; 95%CI, −8.08 to −5.15; *p* < 0.001; Figure 6), IL-6 (SMD = − 4.10; 95%CI, −5.38 to − 2.82; *p* < 0.001; Figure 7), IL-1β (SMD = −4.20; 95%CI, −5.45 to −2.94; *p* < 0.001), CRP (SMD = −5.24; 95%CI, −7.35 to − 3.14; *p* < 0.001), IgG (SMD = − 7.33; 95%CI, −9.81 to −4.84; *p* < 0.001), VEGF (SMD = −8.51; 95%CI, −11.50 to −5.52; *p* < 0.001), and MPO (SMD = − 13.58; 95%CI, −16.98 to −10.18; *p* < 0.001) compared with their levels in nanomaterial-treated animals (Table 3).

Except for IL-1β which was analyzed with a fixed-effects model, other outcomes were analyzed with a random-effects model because of the presence of significant heterogeneity. Thus, subgroup analyses were performed for them. The pooled results showed that, compared with controls, all these pro-inflammatory mediators were still significantly reduced after the administration of flavonoids regardless of which subgroups they belonged to (Supplementary Table 3 and Figures 5–7).

#### Effects of Flavonoid Supplementation on Apoptosis

TUNEL apoptotic index and the mRNA expression levels of apoptosis-related genes (FAS, caspase-3, Bax, and Bcl2) were measured in 2, 7, 7, 12, and 9 datasets, respectively. The pooled analysis of these indicators showed that flavonoid (morin or morin + rutin) supplementation decreased the TUNEL apoptotic index (SMD = − 10.98; 95%CI, −17.63 to –4.33; *p* = 0.001), downregulated the mRNA expression levels of pro-apoptotic factors, such as FAS (SMD = −11.14; 95%CI, −14.01 to −8.27; *p* < 0.001), caspase-3 (SMD = − 10.46; 95%CI, −12.14 to −8.79; *p* < 0.001), and Bax (SMD = − 9.98; 95%CI, −11.73 to −8.23; *p* < 0.001), and upregulated the mRNA expression level of anti-apoptotic Bcl2 (SMD = 9.19; 95%CI, 5.92 – 12.46; *p* < 0.001) compared with the nanomaterial-exposed groups (Table 3). These results of FAS, caspase-3, and Bax indicators were not changed by subgroup factors. The mRNA expression level of Bcl2 seemed to be only significantly increased by quercetin and morin but not by apigenin (Supplementary Table 4).

### Effects of Flavonoid Supplementation on DNA Damage

Comet assay was performed to evaluate the DNA damage in two studies with three datasets, after which the data about tail DNA content and tail length were obtained. The pooled analysis of these data found that flavonoid (quercetin) supplementation led to significant decreases in the tail length (SMD = −13.77; 95%CI, −16.45 to −11.08; *p* < 0.001) and the tail DNA% (SMD = −8.59; 95%CI, −10.31 to −6.87; *p* < 0.001) (Table 3).

### Effects of Flavonoid Supplementation on Body Weight

Eight datasets recorded the body weight of the animals treated with nanomaterials alone or co-administered with flavonoids (Supplementary Table 1). The meta-analysis of these eight datasets under a random-effects model revealed that, relative to nanomaterial-intoxicated animals, flavonoid supplementation significantly increased the body weight of the animals (SMD = 1.11; 95%CI, 0.26–1.97; *p* = 0.011) (Table 3). The subgroup analysis indicated that only morin (*p* = 0.019) and naringenin (*p* = 0.008; this conclusion needed further confirmation because only one study was included) were effective interventions for improving the body weight of animals, but not quercetin (*p* = 0.397). Also, the improvement effect on the body weight was only limited to the subgroups with TiO_2_NP exposure, with flavonoid supplementation for less than 2 weeks and with an intervention dosage of ≤ 100 mg/kg (Supplementary Table 5).

#### Effects of Flavonoid Supplementation on Liver Function

Alanine aminotransferase, AST, ALP, and albumin were determined in our included studies (involving 14, 12, 4, and 4 datasets, respectively) to explore whether flavonoid supplementation could protect against nanomaterial-induced liver injuries (Supplementary Table 1). As shown in Table 3, the levels of ALT (SMD = − 6.15; 95%CI, −8.77 to −3.53; *p* < 0.001), AST (SMD = −5.25; 95%CI, −7.44 to −3.07; *p* < 0.001), and ALP (SMD = −11.26; 95%CI, −17.71 to −4.80; *p* = 0.001) were found to be significantly decreased; the level of albumin (SMD = 8.13; 95%CI, 2.20 – 14.07; *p* = 0.007) was significantly increased in the animals that received flavonoids in addition to nanomaterials.

Subgroup analyses demonstrated that the effects on ALT, AST, and albumin were particularly significant in the subgroups treated with flavonol and flavanone subclasses at a high dosage (>100 mg/kg) or a low dosage (≤50 mg/kg) and undergoing flavonoid treatment for more than 2 weeks. The level of ALP seemed not to be significantly improved in the subgroups stratified by flavonoid subclasses (Supplementary Table 6). Furthermore, flavonoid supplementation prevented ZnONP-, AgNP-, CuONP-, CNT-, and TiO_2_NP-induced increases in the levels of ALT and/or AST and a NiONP-induced increase in the level of albumin (Supplementary Table 6).

#### Effects of Flavonoid Supplementation on Renal Function

Renal function biomarkers (urea, blood urea nitrogen, creatinine, and uric acid) were, respectively, detected in 9, 7, 13, and 7 datasets (Supplementary Table 1). The combined results from a random-effects model indicated that supplementation with flavonoids to the nanomaterial-exposed animals resulted in significant decreases in the levels of urea (SMD = −3.56; 95%CI, −5.08 to −2.04; *p* < 0.001), blood urea nitrogen (SMD = − 5.76; 95%CI, −8.67 to −2.84; *p* < 0.001), creatinine (SMD = −5.22; 95%CI, −7.05 to −3.38; *p* < 0.001), and uric acid (SMD = − 3.63; 95%CI, − 5.68 to −1.58; *p* = 0.001) (Table 3).

Subgroup analyses demonstrated that supplementation with all these three flavonoid subclasses (without dosage requisition) provided statistically significant effects on four renal function biomarkers, especially when the interventions were performed for more than 2 weeks. Additionally, flavonoid supplementation only prevented TiO_2_NP- and AgNP-induced increases in the level of uric acid; the beneficial effects of flavonoids on the levels of urea and blood urea nitrogen were applicable to all studied nanomaterials; MSNP-induced creatinine was not significantly improved by flavonoids (Supplementary Table 7).

#### Effects of Flavonoid Supplementation on Testis Function

Sex hormones (testosterone, FSH, and LH), semen profile (sperm motility, sperm cell count, sperm abnormalities, and live sperm), and steroidogenesis pathway gene (17β-HSD) were reported in 10, 7, 7, 6, 7, 5, 6, and 5 datasets, respectively (Supplementary Table 1). Overall, we found that the intake of flavonoids could trigger marked increases in the serum testosterone level (SMD = 6.97; 95%CI, 4.48 – 9.46; *p* < 0.001), sperm motility (SMD = 7.64; 95%CI, 4.06 – 11.22; *p* < 0.001), cell count (SMD = 5.95; 95%CI, 2.93 – 8.97; *p* < 0.001), live percentage (SMD = 7.05; 95%CI, 1.32 – 12.78; *p* = 0.016), and 17β-HSD expression levels (SMD = 5.35; 95%CI, 3.21 – 7.49; *p* < 0.001) but induce a decrease in the percentage of abnormal sperms (SMD = − 7.73; 95%CI, −9.87 to −5.60; *p* < 0.001) compared with the nanomaterial-exposed group (Table 3). The FSH and LH levels were not significantly changed by flavonoids.

Only the flavonol subclass was investigated in the included studies; thus, the overall meta-analysis results of flavonoid supplementation represented the effects of flavonols on the testis function. Quercetin, rutin, and morin were all shown to significantly improve the testosterone levels, the percentage of abnormal sperms, and the expression levels of 17β-HSD; sperm motility, count, and the percentage of live sperm seemed to be only enhanced by rutin and morin but not by quercetin (Supplementary Table 8). Flavonoid dosage, intervention duration, and route factors did not influence the meta-analysis results (except for live sperm which was only significantly increased when flavonoids were given at a dosage of > 100 mg/kg). Although supplementation with rutin and rutin + morin was shown to increase the FSH and LH levels in the subgroup analyses, there was only one study to demonstrate this conclusion (Supplementary Table 8), and thus, further confirmation was still needed.

#### Effects of Flavonoid Supplementation on Brain Function

Serotonin and AchE were two biomarkers to reflect the nanomaterial-induced neurotoxicity in the brain and the effects of flavonoid supplementation. They were reported in four and ten datasets, respectively. The overall meta-analysis results showed that dietary supplementation with flavonoids was not significantly associated with alterations in the serotonin level (*p* = 0.120). The AChE activity was significantly enhanced by flavonoids (*p* = 0.027) (Table 3), and this significance was amplified in the flavanone (kolaviron) subgroup (*p* < 0.001) (Supplementary Table 9).

### Publication Bias and Sensitivity Analysis

**FIGURE 8:**
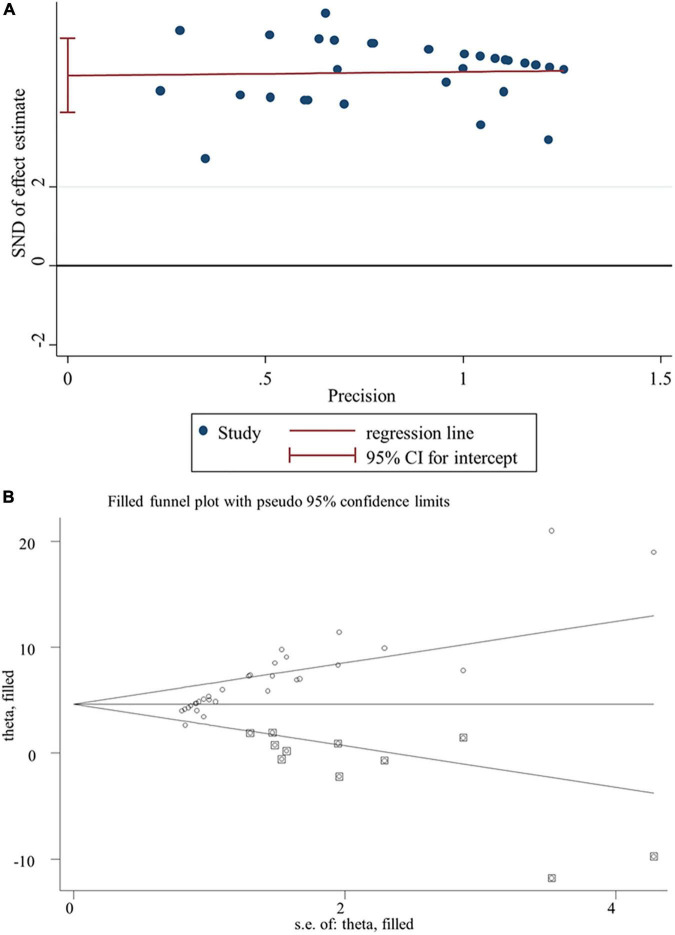
Publication bias analysis for SOD. **(A)** Egger’s publication bias plot; **(B)** the trim-and-fill analysis to adjust estimates. SND, standard normal deviation; CI, confidence interval.

**FIGURE 9:**
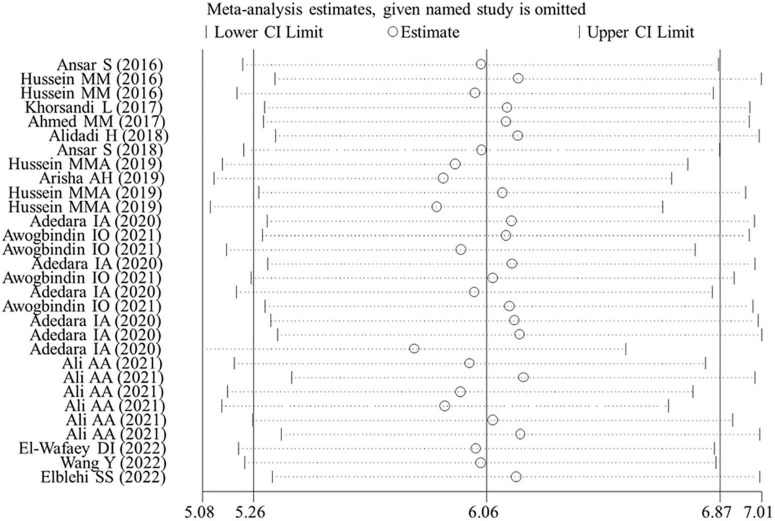
Sensitivity analysis for SOD. CI, confidence interval.

Egger’s linear regression test revealed the presence of the publication bias for the analysis of several indicators (except of GR, *p* = 0.502; FSH, *p* = 0.784; LH, *p* = 0.239; live sperm, *p* = 0.151; caspase-3, *p* = 0.157) (Table 3). Thus, a trim-and-fill method was applied to adjust the effects of the publication bias for these indicators. As a result, beneficial effects of flavonoid supplementation on MDA, NO, TNF-a, IL-6, TNF-α, CRP, IgG, VEGF, MPO, ALT, ALP, AST, urea, blood urea nitrogen, creatinine, uric acid, FAS, Bax mRNA, sperm abnormalities, tail length, and tail DNA% were found not to be altered after correction; significant results were still obtained for SOD (SMD = 4.77; 95%CI, 3.86 – 5.67; *p* < 0.001; Figure 8), CAT (SMD = 4.41; 95%CI, 3.20 – 5.76; *p* < 0.001), GSH (SMD = 3.66; 95%CI, 2.53 – 4.79; *p* < 0.001), GPx (SMD = 1.91; 95%CI, 0.64 – 3.19; *p* < 0.001), GST (SMD = 5.23; 95%CI, 3.53 – 6.94; *p* < 0.001), Bcl2 (SMD = 4.07; 95%CI, 0.77 – 7.37; *p* = 0.016), testosterone (SMD = 2.88; 95%CI, 0.26 – 5.49; *p* = 0.031), sperm motility (SMD = 3.60; 95%CI, 0.17 – 7.03; *p* = 0.04), and 17β-HSD (SMD = 3.58; 95%CI, 1.28 – 5.87; *p* = 0.002); the results of the body weight (SMD = 1.93; 95%CI, 0.77 – 4.86; *p* = 0.160), albumin (SMD = −15.51; 95%CI, −2.85 to 8.34; *p* = 0.337), sperm count (SMD = 2.09; 95%CI, −0.96 to 5.14; *p* = 0.180), live sperm (SMD = 0.83; 95%CI, −4.51 to 6.16; *p* = 0.304), and AChE (SMD = −0.55; 95%CI, −4.42 to 3.31; *p* = 0.780) were no longer significant after correction. The sensitivity analysis results also showed that the exclusion of any single study did not affect the synthesized results (Figure 9).

## Discussion

To our knowledge, this is the first study to systematically evaluate the effects of flavonoid supplementation on nanomaterial-induced toxicities and potential mechanisms. Our meta-analysis of data from 26 preclinical animal studies showed that flavonoid supplementation could significantly increase the levels of anti-oxidative enzymes (SOD, CAT, GSH, GPx, and GST), reduce the production of oxidative agents (MDA) and pro-inflammatory mediators (NO, TNF-α, IL-6, IL-1β, CRP, IgG, VEGF, and MPO), and alleviate cell apoptosis (manifested by decreases in the mRNA expression levels of pro-apoptotic factors, such as caspase-3, FAS, and Bax, and increases in the mRNA expression levels of Bcl2), DNA damage (reductions in tail length and tail DNA%), and nanomaterial-induced injuries of the liver (reduced ALT and AST activities), kidney (reduced urea, blood urea nitrogen, creatinine, and uric acid concentration), testis (increased testosterone, sperm motility, 17β-HSD, and reduced sperm abnormalities), and brain (enhanced AChE activities). Our conclusions were in line with previous meta-analyses that reported the protective roles of flavonoids on other oxidative stress- and inflammation-related diseases (11, 45). These findings suggest that appropriate supplementation of flavonoids may be a potential measure to prevent occupational detriments resulting from nanomaterial exposure.

Eight flavonoid types belonging to three subclasses [flavonols (quercetin, rutin, and morin), flavanones (naringenin, kolaviron, hesperidin, and silibinin), and flavones (apigenin)] were contained in our included studies. As shown in Supplementary Figure 1, all these flavonoids possess phenolic hydroxyl groups structurally. Thus, they can scavenge free radicals directly by donating hydrogen or electron (46) and then inhibit lipid peroxidation to prevent the formation of the end products of lipid peroxidation (MDA) (47). This conclusion was confirmed in our subgroup analyses (i.e., the level of MDA induced by nanomaterials was significantly reduced by flavonoids regardless of flavonoid types, durations, and dosages). Furthermore, flavonoids [e.g., grape seed procyanidin extract (48), apigenin (43), and quercetin (42)] were reported to enhance the expression level of Nrf2, which could subsequently bind with the antioxidant response element to stimulate the transcription of antioxidant enzymes. In accordance with this theory, we found that the levels of SOD, CAT, GSH, GPx, and GST were significantly increased (accompanied by upregulation of Nrf2) after supplementation with overall flavonoids in nanomaterial-intoxicated animals. However, interestingly, subgroup analyses showed that the levels of SOD and CAT changed by various nanomaterials could be influenced by all flavonoid types; morin and hesperidin had no effects on the level of GSH; quercetin did not significantly induce the increase in the level of GPx. These results may be explained by the following reasons: (1) the sample size (especially hesperidin with only two data analyzed) was small, which may cause deviation in the results; (2) SOD and CAT are the first-line enzymes of cellular defense against oxidative injuries, and they can be rapidly induced to decompose O_2_ and H_2_O_2_ to prevent the formation of more reactive hydroxyl radicals (49); and (3) oral quercetin supplementation was found to be superior to intraperitoneal administration (the main route for studies with GPx analyzed, as given in Supplementary Table 1) in the protection against the oxidative stress (50).

There was evidence that nanomaterials caused an inflammatory response (increases in the levels of TNF-α, IL-6, and IL-1β) by triggering the generation of reactive oxygen species (ROS), which then activated mitogen-activated protein kinase–nuclear factor kappa B/activator protein-1 pathways (41, 51–53). Thus, blocking the production of ROS may be a potential reason to explain the anti-inflammatory roles of flavonoid supplementation, which was demonstrated by the study of Lim et al. (41). ChIP-seq analysis identified that Nrf2 directly bound to the upstream regions of pro-inflammatory cytokine genes (IL-6 and IL-1) to inhibit their expressions in M1-type macrophages, which was independent of the redox conditions (54). Therefore, upregulation of Nrf2 expression may be another potential mechanism of flavonoids to alleviate nanomaterial-induced inflammation as demonstrated by Elblehi et al. (42). Moreover, epigenetic regulation was considered to be important for nanomaterial-mediated toxicities (55). Sirtuin 1 (SIRT1), a nicotinamide adenosine dinucleotide-dependent deacetylase, was found to suppress the transcription of high-mobility group box 1 by direct deacetylation regulation (56, 57), which then inhibited cells to release pro-inflammatory mediators (IL-6, TNF-α, CRP, and IgG) by binding to toll-like receptor 4 (57–59). Hereby, the activation of SIRT1 signaling pathways also could protect against nanomaterial-induced inflammation (60). In line with these studies, we found that the levels of TNF-α, IL-6, IL-1β, CRP, and IgG were significantly reduced by flavonoids compared with the nanomaterial-exposed group, and these results were not influenced by subgroup factors.

It had been widely accepted that ROS accumulation induced DNA damage (61) and activated mitochondria-dependent apoptotic signaling pathways (including increasing the expression levels of caspase-3 and Bax and decreasing the expression levels of Bcl2) (62). The addition of pro-inflammatory cytokines TNF-α and IL-1β was demonstrated to increase the Fas expression and Fas-mediated caspase activation and apoptosis (63, 64). Theoretically, the alleviation of oxidative stress and inflammation by flavonoids may correspondingly suppress cell apoptosis and DNA damage. As expected, we found that, compared with the nanomaterial-intoxicated animals, the tail length, tail DNA%, and the mRNA expression levels of caspase-3, Bax, and FAS, as well as the TUNEL apoptotic index, were decreased, and the mRNA expression level of Bcl2 was increased after supplementation with flavonoids. Also, these results on apoptotic indicators were almost not changed by subgroup factors. In addition to apoptosis and DNA damage, previous studies showed that high amounts of NO (65), VEGF (66), and MPO (67) could be synthesized and released in cells in response to ROS and inflammation stimuli. All the enhanced NO (68), VEGF (69), and MPO (70) could mediate an increase in the permeability of vascular endothelium, which may result in the leakage of plasma albumin to tissues and cause hypoxia and edema of organs, ultimately triggering the function dysfunction and exacerbating the disease progression. Thus, NO, VEGF, and MPO were also therapeutic targets to prevent and treat nanomaterial-induced organ injuries. This hypothesis was confirmed by our study which showed that the levels of NO, VEGF, and MPO were significantly reduced by flavonoids. All these alterations in the oxidative stress, inflammation, cell apoptosis, DNA damage, and endothelial dysfunction *via* supplementation with flavonoids may ultimately lead to the attenuation of organ injuries and function failure consequences. As anticipated, our meta-analysis results showed that some liver, kidney, testis, and brain functional biomarkers were significantly improved by flavonoids.

Several limitations should be acknowledged. The first was a limited number of included studies, which led to fewer data available to evaluate the influence on some indicators (e.g., Nrf2, IL-1β, etc.) and no data obtainable to determine the effects of other flavonoid types (except for three subclasses we studied) and other organ injuries. Second, the design was variable for included studies, with different nanomaterial types, subclasses, dosages, intervention durations, routes of flavonoids, sample sources, and animal species, all of which contributed to the presence of considerably high heterogeneity among studies and thus may weaken the robustness of our conclusions. Third, unlike clinical trials where randomization, allocation concealment, and blinding of outcome assessment were mandatory, these details were rarely mentioned in our included animal experiments, resulting in an unclear risk of bias present in the quality assessment. Fourth, some data were extracted from the bar graphs, which may be slightly different from the real data. Accordingly, more experiments and evidence-based studies are needed to confirm the effects of flavonoids on nanomaterial-induced toxicities.

## Conclusion

This meta-analysis suggests that supplementation with flavonoids may be an effective measure to prevent nanomaterial-induced organ injuries by attenuation of oxidative stress, inflammation, apoptosis, DNA damage, and endothelial dysfunction.

## Data Availability Statement

The original contributions presented in this study are included in the article/Supplementary Material, further inquiries can be directed to the corresponding author/s.

## Author Contributions

DX, JH, and XL conceived the idea and designed the study. DX and JH collected the data and performed the statistical analysis. DX wrote the manuscript. TW, WX, QM, and KC contributed to the interpretation of the results. XL revised the manuscript and was the guarantor of the overall content. All authors approved the final version of the manuscript.

## Conflict of Interest

TW was employed by Shanghai Jing Rui Yang Industrial Co., Ltd. WX was employed by Shanghai Nutri-woods Bio-Technology Co., Ltd. QM was employed by Shanghai Pechoin Daily Chemical Co., Ltd. The remaining authors declare that the research was conducted in the absence of any commercial or financial relationships that could be construed as a potential conflict of interest.

## Publisher’s Note

All claims expressed in this article are solely those of the authors and do not necessarily represent those of their affiliated organizations, or those of the publisher, the editors and the reviewers. Any product that may be evaluated in this article, or claim that may be made by its manufacturer, is not guaranteed or endorsed by the publisher.

## Footnotes

1
http://digitizer.sourceforge.net/

## Supplementary Material

Supplementary Figure 1 The structure of each flavonoid.

## Abbreviations

NiONPs: nickel oxide nanoparticles
MDA: malonaldehyde
GSH: glutathione
SOD: superoxide dismutase
AST: aspartate aminotransferase
ALT: alanine aminotransferase
TIO_2_NPs: titanium dioxide nanoparticles
IONP: iron oxide nanoparticle
IL-6: interleukin-6
TNF-α: tumor necrosis factor-α
ZnONP: zinc oxide nanoparticle
PRISMA: preferred reporting items for systematic reviews and meta-analysis
PICOS: participants, interventions, comparisons, outcomes, and study design
GPx: glutathione peroxidase
CAT: catalase
GST: glutathione-S-transferase
GR: glutathione reductase
Nrf2: nuclear factor erythroid 2-related factor 2
CRP: C-reactive protein
IgG: immunoglobulin G
MPO: myeloperoxidase
NO: nitric oxide
VEGF: vascular endothelial growth factor
ALP: alkaline phosphatase
LH: luteinizing hormone
FSH: follicle-stimulating hormone
17β-HSD: 17β-hydroxysteroid dehydrogenase type
AChE: acetylcholinesterase
FAS: Fas cell surface death receptor
Bcl2: B-cell lymphoma-2
Bax: BCL2-associated X, apoptosis regulator
SMD: standardized mean difference
CI: confidence interval
AgNPs: silver nanoparticles
CNTs: carbon nanotubes
CuONPs: copper oxide nanoparticles
GNPs: gold nanoparticles
SiONPs: silica dioxide nanoparticles
MSNPs: mesoporous silica nanoparticles
ROS: reactive oxygen species
SIRT1: sirtuin 1.
