# Supplementary material for: Effects of Flavonoid Supplementation on Nanomaterial-Induced Toxicity: A Meta-Analysis of Preclinical Animal Studies
Source: Front Nutr. 2022 Jun 14;9:929343. doi: 10.3389/fnut.2022.929343 (PMC9237539; doi:10.3389/fnut.2022.929343)
Supplement: Supplementary file 3 [file Table_2.DOCX]

**Supplementary table 2 Subgroup results for oxidative stress indicators**

|  | Variables | No. | SMD | 95%CI | P_E_-value | I^2^ | P_H_-value | Model |
| --- | --- | --- | --- | --- | --- | --- | --- | --- |
| **MDA** | Nanomaterial types |  |  |  |  |  |  |  |
|  | TiO_2_NPs | 20 | -6.03 | -7.58,-4.49 | **<0.001** | 88.8 | <0.001 | R |
|  | ZnONPs | 5 | -4.93 | -7.00,-2.87 | **<0.001** | 79.7 | 0.001 | R |
|  | AgNPs | 3 | -8.30 | -9.92,-6.67 | **<0.001** | 0.0 | 0.452 | F |
|  | CNTs | 10 | -10.39 | -12.93,-7.86 | **<0.001** | 83.7 | <0.001 | R |
|  | GNPs | 2 | -4.62 | -6.43,-2.81 | **<0.001** | 5.4 | 0.304 | F |
|  | IONPs | 3 | -4.28 | -6.12,-2.43 | **<0.001** | 64.1 | 0.062 | R |
|  | NiONPs | 6 | -9.81 | -15.27,-4.34 | **<0.001** | 93.2 | <0.001 | R |
|  | SiO_2_NPs | 1 | -7.90 | -13.61,-2.20 | 0.007 | - | - | R |
|  | Flavonoid subclasses |  |  |  |  |  |  |  |
|  | Flavonols | 30 | -5.96 | -7.13,-4.80 | **<0.001** | 87.2 | <0.001 | R |
|  | (Quercetin) | 19 | -4.60 | -5.70,-3.50 | **<0.001** | 81.2 | <0.001 | R |
|  | (Rutin) | 2 | -8.11 | -10.14,-6.08 | **<0.001** | 0.0 | 0.657 | R |
|  | (Morin) | 8 | -8.45 | -12.29,-4.61 | **<0.001** | 92.3 | <0.001 | R |
|  | (Morin + rutin) | 1 | -13.96 | -18.61,-9.32 | <0.001 | - | - | R |
|  | Flavanones | 13 | -6.92 | -8.95,-11.29 | **<0.001** | 89.3 | <0.001 | R |
|  | (Hesperidin) | 2 | -5.53 | -7.86,-3.20 | **<0.001** | 29.7 | 0.233 | R |
|  | (Kolaviron) | 10 | -10.39 | -12.93,-7.86 | **<0.001** | 83.7 | <0.001 | R |
|  | (Naringenin) | 1 | -2.06 | -3.30,-0.82 | 0.001 | - | - | R |
|  | Flavones | 7 | -9.48 | -14.43,-4.54 | **<0.001** | 92.3 | <0.001 | R |
|  | (Apigenin) | 7 | -9.48 | -14.43,-4.54 | **<0.001** | 92.3 | <0.001 | R |
|  | Flavonoid dosage |  |  |  |  |  |  |  |
|  | ≤ 50 mg/kg | 32 | -7.33 | -8.79,-5.86 | **<0.001** | 90.1 | <0.001 | R |
|  | ≤100 mg/kg | 15 | -6.53 | -8.20,-4.86 | **<0.001** | 88.1 | <0.001 | R |
|  | > 100 mg/kg | 3 | -7.27 | -12.72,-1.82 | **0.009** | 90.5 | <0.001 | R |
|  | Intervention duration |  |  |  |  |  |  |  |
|  | ≤2 weeks | 17 | -6.13 | -7.80,-4.46 | **<0.001** | 85.5 | <0.001 | R |
|  | ≤4 weeks | 21 | -8.01 | -9.94,-6.09 | **<0.001** | 91.7 | <0.001 | R |
|  | > 4 weeks | 12 | -6.85 | -8.74,-4.97 | **<0.001** | 88.3 | <0.001 | R |
|  | Flavonoid route |  |  |  |  |  |  |  |
|  | Orally | 28 | -7.62 | -9.07,-6.17 | **<0.001** | 89.5 | <0.001 | R |
|  | Intraperitoneally | 10 | -4.87 | -6.82,-2.92 | **<0.001** | 83.0 | <0.001 | R |
|  | Intragastrically | 12 | -7.61 | -10.07,-5.15 | **<0.001** | 91.6 | <0.001 | R |
|  | Sample source |  |  |  |  |  |  |  |
|  | Brain | 12 | -7.88 | -9.71,-6.05 | **<0.001** | 81.7 | <0.001 | R |
|  | Liver | 13 | -4.03 | -5.49,-2.56 | **<0.001** | 86.9 | <0.001 | R |
|  | Kidney | 11 | -9.86 | -12.81,-6.90 | **<0.001** | 86.1 | <0.001 | R |
|  | Prostate | 3 | -9.43 | -14.20,-4.66 | **<0.001** | 71.6 | 0.030 | R |
|  | Testis | 10 | -7.06 | -9.62,-4.50 | **<0.001** | 92.5 | <0.001 | R |
|  | Lung | 1 | -7.90 | -13.61,-2.20 | 0.007 | - | - | R |
|  | Animal species |  |  |  |  |  |  |  |
|  | Mice | 2 | -4.10 | -10.16,-1.96 | 0.184 | 77.9 | 0.033 | R |
|  | Rats | 48 | -7.16 | -8.25,-6.07 | **<0.001** | 89.2 | <0.001 | R |
| **SOD** | Nanomaterial types |  |  |  |  |  |  |  |
|  | TiO_2_NPs | 5 | 6.49 | 4.29,8.69 | **<0.001** | 76.9 | 0.002 | R |
|  | ZnONPs | 5 | 6.22 | 4.64,7.80 | **<0.001** | 46.3 | 0.114 | **F** |
|  | AgNPs | 3 | 6.10 | 3.33,8.87 | **<0.001** | 80.2 | **0.006** | R |
|  | CNTs | 10 | 5.77 | 4.51,7.02 | **<0.001** | 72.0 | <0.001 | R |
|  | NiONPs | 6 | 6.96 | 3.80,9.51 | **<0.001** | 82.3 | <0.001 | R |
|  | SiO_2_NPs | 1 | 7.79 | 2.16,13.43 | 0.007 | - | **-** | R |
|  | Flavonoid subclasses |  |  |  |  |  |  |  |
|  | Flavonols | 10 | 6.07 | 4.77,7.37 | **<0.001** | 72.0 | <0.001 | R |
|  | (Quercetin) | 5 | 4.65 | 3.70,5.61 | **<0.001** | 23.6 | 0.264 | **F** |
|  | (Rutin) | 2 | 5.17 | 3.78,6.55 | **<0.001** | 0.0 | 0.803 | **F** |
|  | (Morin) | 2 | 10.42 | 8.06,12.79 | **<0.001** | 0.0 | 0.509 | **F** |
|  | (Morin + rutin) | 1 | 9.04 | 5.96,12.12 | <0.001 | - | - | R |
|  | Flavanones | 13 | 5.98 | 4.89,7.07 | **<0.001** | 66.7 | <0.001 | R |
|  | (Hesperidin) | 2 | 6.95 | 4.65,9.24 | **<0.001** | 0.0 | 0.968 | **F** |
|  | (Kolaviron) | 10 | 5.77 | 4.51,7.02 | **<0.001** | 72.0 | <0.001 | R |
|  | (Naringenin) | 1 | 7.29 | 4.42,10.16 | 0.001 | - | - | R |
|  | Flavones | 7 | 6.75 | 4.10,9.41 | **<0.001** | 79.7 | <0.001 | R |
|  | (Apigenin) | 7 | 6.75 | 4.10,9.41 | **<0.001** | 79.7 | <0.001 | R |
|  | Flavonoid dosage |  |  |  |  |  |  |  |
|  | ≤ 50 mg/kg | 16 | 5.82 | 4.68,6.95 | **<0.001** | 72.7 | <0.001 | R |
|  | ≤ 100 mg/kg | 13 | 6.15 | 5.00,7.31 | **<0.001** | 68.0 | <0.001 | R |
|  | > 100 mg/kg | 1 | 9.04 | 5.96,12.12 | 0.009 | - | - | R |
|  | Intervention duration |  |  |  |  |  |  |  |
|  | ≤ 2 weeks | 7 | 5.56 | 3.81,7.32 | **<0.001** | 68.6 | 0.004 | R |
|  | ≤ 4 weeks | 14 | 6.19 | 4.94,7.44 | **<0.001** | 73.6 | <0.001 | R |
|  | > 4 weeks | 9 | 6.36 | 4.93,7.79 | **<0.001** | 72.8 | <0.001 | R |
|  | Flavonoid route |  |  |  |  |  |  |  |
|  | Orally | 24 | 5.94 | 5.04,6.85 | **<0.001** | 71.1 | <0.001 | R |
|  | Intragastrically | 6 | 6.58 | 4.72,8.44 | **<0.001** | 73.7 | 0.002 | R |
|  | Sample source |  |  |  |  |  |  |  |
|  | Brain | 8 | 5.49 | 4.18,6.81 | **<0.001** | 71.6 | <0.001 | R |
|  | Liver | 7 | 5.59 | 4.32,6.87 | **<0.001** | 46.1 | 0.084 | F |
|  | Kidney | 6 | 6.54 | 3.97,9.10 | **<0.001** | 83.0 | <0.001 | R |
|  | Testis | 7 | 7.04 | 5.17,8.92 | **<0.001** | 76.4 | <0.001 | R |
|  | Lung | 1 | 7.79 | 2.16,13.43 | 0.007 | - | - | R |
|  | Animal species |  |  |  |  |  |  |  |
|  | Mice | 2 | 5.20 | 3.27,7.12 | <0.001 | 0.0 | 0.336 | F |
|  | Rats | 28 | 6.11 | 5.27,6.96 | **<0.001** | 72.8 | <0.001 | R |
| **CAT** | Nanomaterial types |  |  |  |  |  |  |  |
|  | TiO_2_NPs | 5 | 5.35 | 2.98,7.73 | **<0.001** | 86.6 | <0.001 | R |
|  | ZnONPs | 4 | 4.86 | 2.65,7.08 | **<0.001** | 76.9 | 0.005 | R |
|  | AgNPs | 3 | 7.35 | 4.36,10.35 | **<0.001** | 75.9 | 0.016 | R |
|  | CNTs | 10 | 8.05 | 6.22,9.89 | **<0.001** | 79.3 | <0.001 | R |
|  | Flavonoid subclasses |  |  |  |  |  |  |  |
|  | Flavonols | 10 | 5.75 | 4.11,7.38 | **<0.001** | 85.6 | <0.001 | R |
|  | (Quercetin) | 5 | 3.78 | 2.36,5.20 | **<0.001** | 74.8 | 0.003 | R |
|  | (Rutin) | 2 | 6.68 | 4.95,8.41 | **<0.001** | 0.8 | 0.315 | F |
|  | (Morin) | 2 | 7.31 | 2.67,11.95 | **0.002** | 85.4 | 0.009 | R |
|  | (Morin + rutin) | 1 | 13.66 | 9.11,18.21 | <0.001 | - | - | R |
|  | Flavanones | 12 | 7.47 | 5.94,9.01 | **<0.001** | 75.7 | <0.001 | R |
|  | (Hesperidin) | 2 | 5.28 | 3.46,7.11 | **<0.001** | 0.0 | 0.967 | F |
|  | (Kolaviron) | 10 | 8.05 | 6.22,9.89 | **<0.001** | 79.3 | <0.001 | R |
|  | Flavonoid dosage |  |  |  |  |  |  |  |
|  | ≤ 50 mg/kg | 9 | 6.79 | 5.27,8.31 | **<0.001** | 73.5 | <0.001 | R |
|  | ≤100 mg/kg | 12 | 6.11 | 4.51,7.71 | **<0.001** | 85.1 | <0.001 | R |
|  | > 100 mg/kg | 1 | 13.66 | 9.11,18.21 | 0.009 | - | - | R |
|  | Intervention duration |  |  |  |  |  |  |  |
|  | ≤ 2 weeks | 2 | 5.28 | 3.46,7.11 | **<0.001** | 0.0 | 0.967 | F |
|  | ≤ 4 weeks | 11 | 7.57 | 5.79,9.34 | **<0.001** | 82.2 | <0.001 | R |
|  | > 4 weeks | 9 | 6.10 | 4.24,7.96 | **<0.001** | 86.9 | <0.001 | R |
|  | Flavonoid route |  |  |  |  |  |  |  |
|  | Orally | 16 | 7.13 | 5.83,8.43 | **<0.001** | 77.2 | <0.001 | R |
|  | Intragastrically | 6 | 5.45 | 3.28,-7.63 | **<0.001** | 87.5 | <0.001 | R |
|  | Sample source |  |  |  |  |  |  |  |
|  | Brain | 9 | 7.89 | 5.86,9.92 | **<0.001** | 80.8 | <0.001 | R |
|  | Liver | 3 | 6.19 | 4.44,7.94 | **<0.001** | 35.1 | 0.214 | F |
|  | Kidney | 3 | 5.65 | 2.84,8.46 | **<0.001** | 80.9 | 0.005 | R |
|  | Testis | 7 | 6.11 | 3.86,8.36 | **<0.001** | 89.0 | <0.001 | R |
|  | Animal species |  |  |  |  |  |  |  |
|  | Mice | 1 | 2.24 | 0.96,3.52 | 0.001 | - | - | R |
|  | Rats | 21 | 6.92 | 5.75,8.10 | **<0.001** | 81.0 | <0.001 | R |
| **GSH** | Nanomaterial types |  |  |  |  |  |  |  |
|  | TiO_2_NPs | 11 | 5.13 | 2.14,8.11 | **0.001** | 89.9 | <0.001 | R |
|  | ZnONPs | 6 | 5.07 | 2.79,7.35 | **<0.001** | 88.6 | <0.001 | R |
|  | AgNPs | 2 | 4.71 | 3.02,6.40 | <0.001 | 40.3 | 0.195 | F |
|  | CNTs | 10 | 7.05 | 5.12,8.98 | **<0.001** | 86.9 | <0.001 | R |
|  | GNPs | 2 | 830.44 | -385.58,2046.46 | 0.181 | 92.5 | <0.001 | R |
|  | IONPs | 3 | 3.23 | 2.10,4.35 | **<0.001** | 34.2 | 0.219 | R |
|  | NiONPs | 6 | 5.11 | 2.50,7.28 | **<0.001** | 87.1 | <0.001 | R |
|  | Flavonoid subclasses |  |  |  |  |  |  |  |
|  | Flavonols | 22 | 4.93 | 3.38,6.48 | **<0.001** | 89.5 | <0.001 | R |
|  | (Quercetin) | 12 | 4.58 | 2.96,6.20 | **<0.001** | 87.2 | <0.001 | R |
|  | (Rutin) | 2 | 5.36 | 2.34,8.38 | **0.001** | 76.1 | 0.041 | R |
|  | (Morin) | 7 | 2.65 | -3.23,8.53 | 0.377 | 93.4 | <0.001 | R |
|  | (Morin + rutin) | 1 | 8.92 | 5.87,11.96 | <0.001 | - | - | R |
|  | Flavanones | 12 | 7.04 | 5.22,8.85 | **<0.001** | 86.8 | <0.001 | R |
|  | (Hesperidin) | 2 | 9.56 | -4.29,23.41 | 0.176 | 92.1 | <0.001 | R |
|  | (Kolaviron) | 10 | 7.05 | 5.12,8.98 | **<0.001** | 86.9 | <0.001 | R |
|  | Flavones | 6 | 5.11 | 2.50,7.73 | **<0.001** | 87.1 | <0.001 | R |
|  | (Apigenin) | 6 | 5.11 | 2.50,7.73 | **<0.001** | 87.1 | <0.001 | R |
|  | Flavonoid dosage |  |  |  |  |  |  |  |
|  | ≤ 50 mg/kg | 22 | 4.92 | 3.53,6.31 | **<0.001** | 88.2 | <0.001 | R |
|  | ≤100 mg/kg | 12 | 6.39 | 4.34,8.43 | **<0.001** | 90.0 | <0.001 | R |
|  | > 100 mg/kg | 6 | 7.18 | 4.38,9.98 | **<0.001** | 82.5 | <0.001 | R |
|  | Intervention duration |  |  |  |  |  |  |  |
|  | ≤ 2 weeks | 12 | 4.20 | 1.56,6.84 | **0.002** | 90.6 | <0.001 | R |
|  | ≤ 4 weeks | 18 | 7.14 | 5.56,8.73 | **<0.001** | 85.9 | <0.001 | R |
|  | > 4 weeks | 10 | 4.69 | 3.28,6.09 | **<0.001** | 84.8 | <0.001 | R |
|  | Flavonoid route |  |  |  |  |  |  |  |
|  | Orally | 27 | 5.72 | 4.69,6.74 | **<0.001** | 84.5 | <0.001 | R |
|  | Intraperitoneally | 2 | 830.44 | -385.58,2046.46 | 0.181 | 92.5 | <0.001 | R |
|  | Intragastrically | 11 | 4.46 | 1.67,7.24 | **0.002** | 92.4 | <0.001 | R |
|  | Sample source |  |  |  |  |  |  |  |
|  | Brain | 12 | 5.98 | 4.40,7.55 | **<0.001** | 85.2 | <0.001 | R |
|  | Liver | 7 | 5.93 | 2.83,9.03 | **<0.001** | 90.4 | <0.001 | R |
|  | Kidney | 10 | 5.79 | 3.94,7.63 | **<0.001** | 83.2 | <0.001 | R |
|  | Prostate | 3 | 8.45 | 6.18,10.72 | **<0.001** | 0.0 | 0.383 | R |
|  | Testis | 8 | 2.83 | -0.38,6.05 | 0.084 | 93.6 | <0.001 | R |
| **GPx** | Nanomaterial types |  |  |  |  |  |  |  |
|  | TiO_2_NPs | 8 | -0.26 | -1.56,1.04 | 0.694 | 82.4 | <0.001 | R |
|  | ZnONPs | 4 | 3.62 | 1.38,5.86 | **0.002** | 86.1 | <0.001 | R |
|  | AgNPs | 3 | 4.34 | 1.59,7.08 | **0.002** | 83.8 | 0.002 | R |
|  | CNTs | 10 | 6.87 | 5.00,8.74 | **<0.001** | 86.4 | <0.001 | R |
|  | Flavonoid subclasses |  |  |  |  |  |  |  |
|  | Flavonols | 13 | 1.25 | -0.01,2.50 | 0.051 | 89.5 | <0.001 | R |
|  | (Quercetin) | 11 | 0.69 | -0.57,1.84 | 0.286 | 87.9 | <0.001 | R |
|  | (Rutin) | 1 | 5.91 | 3.66,8.16 | <0.001 | - | - | R |
|  | (Morin) | 1 | 3.09 | 1.88,4.31 | <0.001 | - | - | R |
|  | Flavanones | 12 | 6.81 | 5.15,8.47 | **<0.001** | 84.1 | <0.001 | R |
|  | (Hesperidin) | 2 | 6.46 | 4.28,8.64 | **<0.001** | 0.0 | 0.317 | R |
|  | (Kolaviron) | 10 | 6.87 | 5.00,8.74 | **<0.001** | 86.4 | <0.001 | R |
|  | Flavonoid dosage |  |  |  |  |  |  |  |
|  | ≤ 50 mg/kg | 16 | 2.57 | 1.10,4.04 | **0.001** | 92.0 | <0.001 | R |
|  | ≤100 mg/kg | 9 | 6.24 | 4.01,8.46 | **<0.001** | 91.4 | <0.001 | R |
|  | Intervention duration |  |  |  |  |  |  |  |
|  | ≤ 2 weeks | 6 | 1.81 | -0.72,4.34 | 0.162 | 90.0 | <0.001 | R |
|  | ≤ 4 weeks | 14 | 4.76 | 2.81,6.71 | **<0.001** | 93.5 | <0.001 | R |
|  | > 4 weeks | 5 | 3.71 | 1.84,5.58 | **<0.001** | 88.4 | <0.001 | R |
|  | Flavonoid route |  |  |  |  |  |  |  |
|  | Orally | 15 | 6.45 | 5.09,7.82 | **<0.001** | 83.1 | <0.001 | R |
|  | Intraperitoneally | 8 | -0.26 | -1.56,1.04 | 0.694 | 82.4 | <0.001 | R |
|  | Intragastrically | 2 | 1.61 | 0.35,2.88 | **0.012** | 65.4 | 0.089 | R |
|  | Sample source |  |  |  |  |  |  |  |
|  | Brain | 9 | 7.50 | 5.41,9.59 | **<0.001** | 83.6 | <0.001 | R |
|  | Liver | 7 | 2.46 | 0.90,4.02 | **0.002** | 85.0 | <0.001 | R |
|  | Kidney | 6 | 1.09 | -2.03,4.20 | 0.494 | 94.2 | <0.001 | R |
|  | Testis | 3 | 2.09 | 0.85,3.32 | **0.001** | 73.7 | 0.022 | R |
| **GST** | Flavonoid dosage |  |  |  |  |  |  |  |
|  | ≤ 50 mg/kg | 5 | 5.73 | 3.79,7.66 | **<0.001** | 77.8 | 0.001 | R |
|  | ≤100 mg/kg | 5 | 8.29 | 6.13,10.45 | **<0.001** | 64.9 | 0.022 | R |
|  | Sample source |  |  |  |  |  |  |  |
|  | Brain | 6 | 8.03 | 5.07,11.00 | **<0.001** | 87.5 | <0.001 | R |
|  | Liver | 2 | 5.50 | 3.87,7.13 | **<0.001** | 22.6 | 0.256 | F |
|  | Kidney | 2 | 6.36 | 4.76,7.96 | **<0.001** | 0.0 | 0.499 | F |
| **GR** | Nanomaterial types |  |  |  |  |  |  |  |
|  | TiO_2_NPs | 8 | 1.35 | -0.69,3.39 | 0.195 | 88.3 | <0.001 | R |
|  | ZnONPs | 1 | 3.68 | 1.71,5.65 | <0.001 | - | - | R |
|  | Flavonoid subclasses |  |  |  |  |  |  |  |
|  | Flavonols | 8 | 1.35 | -0.69,3.39 | 0.195 | 88.3 | <0.001 | R |
|  | (Quercetin) | 8 | 1.35 | -0.69,3.39 | 0.195 | 88.3 | <0.001 | R |
|  | Flavanones | 1 | 3.68 | 1.71,5.65 | <0.001 | - | - | R |
|  | (Hesperidin) | 1 | 3.68 | 1.71,5.65 | <0.001 | - | - | R |
|  | Flavonoid dosage |  |  |  |  |  |  |  |
|  | ≤ 50 mg/kg | 8 | 1.35 | -0.69,3.39 | 0.195 | 88.3 | <0.001 | R |
|  | ≤100 mg/kg | 1 | 3.68 | 1.71,5.65 | <0.001 | - | - | R |
|  | Intervention duration |  |  |  |  |  |  |  |
|  | ≤ 2 weeks | 5 | 3.95 | 1.38,6.51 | 0.003 | 84.6 | <0.001 | R |
|  | ≤ 4 weeks | 4 | -1.12 | -3.93,1.69 | 0.436 | 89.5 | <0.001 | R |
|  | Flavonoid route |  |  |  |  |  |  |  |
|  | Orally | 1 | 3.68 | 1.71,5.65 | <0.001 | - | - | R |
|  | Intraperitoneally | 8 | 1.35 | -0.69,3.39 | 0.195 | 88.3 | <0.001 | R |
|  | Sample source |  |  |  |  |  |  |  |
|  | Brain | 1 | 3.68 | 1.71,5.65 | <0.001 | - | - | R |
|  | Liver | 4 | 1.23 | -1.16,3.61 | 0.313 | 86.7 | <0.001 | R |
|  | Kidney | 4 | 0.91 | -2.89,4.70 | 0.640 | 89.4 | <0.001 | R |

TiO_2_NPs, titanium dioxide nanoparticles; CuONPs, copper oxide nanoparticles; IONPs, iron oxide nanoparticles; ZnONPs, zinc oxide nanoparticles; GNPs, gold nanoparticles; NiONPs, nickel oxide nanoparticles; AgNPs, silver nanoparticles; CNTs, carbon nanotubes; SiONPs, silica dioxide nanoparticles; MDA, malonaldehyde; SOD, superoxide dismutase; GSH, glutathione; GPx, glutathione peroxidase; CAT, catalase; GST, Glutathione-S-transferase; GR, glutathione reductase; SMD, standardized mean difference; CI, confidence interval; F, fixed-effects; R, random-effects; P_H_-value, significance for heterogeneity; P_E_-value, significance for treatment effects. Bold indicated the outcomes significantly changed by flavonoids (analysis with at least two datasets).
