# Supplementary material for: Effects of Flavonoid Supplementation on Nanomaterial-Induced Toxicity: A Meta-Analysis of Preclinical Animal Studies
Source: Front Nutr. 2022 Jun 14;9:929343. doi: 10.3389/fnut.2022.929343 (PMC9237539; doi:10.3389/fnut.2022.929343)
Supplement: Supplementary file 4 [file Table_3.DOCX]

**Supplementary table 3 Subgroup results for inflammation indicators**

|  | Studies | No. | SMD | 95%CI | P_E_-value | I^2^ | P_H_-value | Model |
| --- | --- | --- | --- | --- | --- | --- | --- | --- |
| NO | Nanomaterial types |  |  |  |  |  |  |  |
|  | TiO_2_NPs | 3 | -10.43 | -13.28,-7.58 | **<0.001** | 0.0 | 0.815 | F |
|  | ZnONPs | 3 | -10.69 | -12.88,-8.51 | **<0.001** | 47.3 | 0.150 | F |
|  | CNTs | 10 | -10.84 | -13.92,-7.77 | **<0.001** | 90.1 | <0.001 | R |
|  | Flavonoid subclasses |  |  |  |  |  |  |  |
|  | Flavonols | 6 | -10.31 | -11.74,-8.87 | **<0.001** | 0.0 | 0.510 | F |
|  | (Quercetin) | 6 | -10.31 | -11.74,-8.87 | **<0.001** | 0.0 | 0.510 | F |
|  | Flavanones | 10 | -10.84 | -13.92,-7.77 | **<0.001** | 90.1 | <0.001 | R |
|  | (Kolaviron) | 10 | -10.84 | -13.92,-7.77 | **<0.001** | 90.1 | <0.001 | R |
|  | Flavonoid dosage |  |  |  |  |  |  |  |
|  | ≤ 50 mg/kg | 5 | -9.52 | -13.54,-5.51 | **<0.001** | 88.5 | <0.001 | R |
|  | ≤100 mg/kg | 5 | -12.68 | -19.00,-6.36 | **<0.001** | 92.9 | <0.001 | R |
|  | > 100 mg/kg | 6 | -10.31 | -11.74,-8.87 | **<0.001** | 0.0 | 0.510 | F |
|  | Sample source |  |  |  |  |  |  |  |
|  | Brain | 6 | -13.53 | -16.72,-10.34 | **<0.001** | 67.0 | 0.010 | R |
|  | Liver | 2 | -3.86 | -4.95,-2.78 | **<0.001** | 0.0 | 0.940 | F |
|  | Kidney | 2 | -10.51 | -13.03,-7.99 | **<0.001** | 0.0 | 0.359 | F |
|  | Serum | 6 | -10.31 | -11.74,-8.87 | **<0.001** | 0.0 | 0.510 | F |
| TNF-α | Nanomaterial types |  |  |  |  |  |  |  |
|  | TiO_2_NPs | 9 | -6.74 | -8.90,-4.58 | **<0.001** | 83.4 | <0.001 | R |
|  | ZnONPs | 5 | -4.18 | -7.68,-0.68 | **0.019** | 95.5 | <0.001 | R |
|  | CNTs | 10 | -8.29 | -9.73,-6.84 | **<0.001** | 61.4 | 0.006 | R |
|  | SiONPs | 2 | -2.57 | -3.70,-1.44 | **<0.001** | 0.0 | 0.729 | F |
|  | AgNPs | 1 | -8.89 | -11.93,-5.86 | <0.001 | - | - | R |
|  | MSNPs | 1 | -3.68 | -6.70,-0.67 | 0.017 | - | - | R |
|  | Flavonoid subclasses |  |  |  |  |  |  |  |
|  | Flavonols | 13 | -7.09 | -9.74,-4.43 | **<0.001** | 93.8 | <0.001 | R |
|  | (Quercetin) | 7 | -5.89 | -9.42,-2.36 | **0.001** | 95.8 | <0.001 | R |
|  | (Morin) | 6 | -8.10 | -10.27,-5.92 | **<0.001** | 48.7 | 0.083 | R |
|  | Flavanones | 14 | -6.47 | -8.28,-4.66 | **<0.001** | 90.1 | <0.001 | R |
|  | (Hesperidin) | 1 | -1.84 | -3.23,-0.45 | 0.009 | - | - | R |
|  | (Kolaviron) | 10 | -8.29 | -9.73,-6.84 | **<0.001** | 61.4 | 0.006 | R |
|  | (Naringenin) | 1 | -1.25 | -2.34,-0.17 | 0.023 | - | - | R |
|  | (Silibinin) | 2 | -2.57 | -3.70,-1.44 | **<0.001** | 0.0 | 0.729 | F |
|  | Flavones | 1 | -3.68 | -6.70,-0.67 | 0.017 | - | - | R |
|  | (Apigenin) | 1 | -3.68 | -6.70,-0.67 | 0.017 | - | - | R |
|  | Flavonoid dosage |  |  |  |  |  |  |  |
|  | ≤ 50 mg/kg | 15 | -6.71 | -8.19,-5.23 | **<0.001** | 77.5 | <0.001 | R |
|  | ≤100 mg/kg | 7 | -7.26 | -10.48,-4.03 | **<0.001** | 93.3 | <0.001 | R |
|  | > 100 mg/kg | 6 | -5.38 | -9.07,-1.68 | **<0.001** | 96.0 | <0.001 | R |
|  | Intervention duration |  |  |  |  |  |  |  |
|  | ≤ 2 weeks | 9 | -4.29 | -5.96,-2.62 | **<0.001** | 81.6 | <0.001 | R |
|  | ≤ 4 weeks | 18 | -7.43 | -9.59,-5.28 | **<0.001** | 83.7 | <0.001 | R |
|  | > 4 weeks | 1 | -8.89 | -11.93,-5.86 | **<0.001** | - | - | R |
|  | Flavonoid route |  |  |  |  |  |  |  |
|  | Orally | 21 | -6.28 | -7.95,-4.61 | **<0.001** | 93.2 | <0.001 | R |
|  | Intragastrically | 6 | -8.10 | -10.27,-5.92 | **<0.001** | 48.7 | 0.083 | R |
|  | Intraperitoneally | 1 | -3.68 | -6.70,-0.67 | 0.017 | - | - | R |
|  | Sample source |  |  |  |  |  |  |  |
|  | Brain | 7 | -8.83 | -12.46,-5.21 | **<0.001** | 91.2 | <0.001 | R |
|  | Liver | 3 | -6.07 | -8.43,-3.71 | **0.018** | 91.9 | <0.001 | R |
|  | Kidney | 3 | -6.99 | -9.09,-4.90 | **<0.001** | 57.7 | 0.094 | R |
|  | Prostate | 3 | -8.16 | -10.64-5.69 | **<0.001** | 19.5 | 0.289 | F |
|  | Testis | 3 | -8.31 | -12.50,-4.13 | **<0.001** | 70.5 | 0.034 | R |
|  | Serum | 7 | -5.89 | -9.42,-2.36 | **0.001** | 95.8 | <0.001 | R |
|  | BALF | 2 | -2.57 | -3.70,-1.44 | **<0.001** | 0.0 | 0.729 | F |
|  | Animal species |  |  |  |  |  |  |  |
|  | Mice | 3 | -2.71 | -3.77,-1.65 | **<0.001** | 0.0 | 0.748 | F |
|  | Rats | 25 | -7.15 | -8.82,-5.47 | **<0.001** | 92.7 | <0.001 | R |
| IL-6 | Nanomaterial types |  |  |  |  |  |  |  |
|  | TiO_2_NPs | 3 | -3.60 | -5.74,-1.47 | **0.001** | 83.4 | 0.002 | R |
|  | ZnONPs | 4 | -3.62 | -5.85,-1.39 | **0.001** | 88.4 | <0.001 | R |
|  | SiONPs | 2 | -4.62 | -6.25,-2.98 | **<0.001** | 0.0 | 0.935 | F |
|  | AgNPs | 1 | -9.12 | -12.23,-6.02 | <0.001 | - | - | R |
|  | MSNPS | 1 | -3.11 | -5.80,-0.43 | 0.023 | - | - | R |
|  | Flavonoid subclasses |  |  |  |  |  |  |  |
|  | Flavonols | 7 | -4.61 | -6.41,-2.81 | **<0.001** | 88.8 | <0.001 | R |
|  | (Quercetin) | 7 | -4.61 | -6.41,-2.81 | **<0.001** | 88.8 | <0.001 | R |
|  | Flavanones | 3 | -3.48 | -5.62,-1.34 | **<0.001** | 71.6 | 0.029 | R |
|  | (Hesperidin) | 1 | -1.73 | -3.09,-0.37 | 0.013 | - | - | R |
|  | (Silibinin) | 2 | -4.62 | -6.25,-2.98 | **<0.001** | 0.0 | 0.935 | F |
|  | Flavones | 1 | -3.11 | -5.80,-0.43 | 0.023 | - | - | R |
|  | (Apigenin) | 1 | -3.11 | -5.80,-0.43 | 0.023 | - | - | R |
|  | Flavonoid dosage |  |  |  |  |  |  |  |
|  | ≤ 50 mg/kg | 4 | -5.23 | -7.45,-3.01 | **<0.001** | 66.2 | 0.031 | R |
|  | ≤100 mg/kg | 1 | -1.73 | -3.09,-0.37 | 0.013 | - | - | R |
|  | > 100 mg/kg | 6 | -3.90 | -5.57,-2.23 | **<0.001** | 86.8 | <0.001 | R |
|  | Intervention duration |  |  |  |  |  |  |  |
|  | ≤ 2 weeks | 4 | -3.34 | -4.96,-1.73 | **0.001** | 57.6 | 0.070 | R |
|  | ≤ 4 weeks | 6 | -3.90 | -5.57,-2.23 | **<0.001** | 86.8 | <0.001 | R |
|  | > 4 weeks | 1 | -9.12 | -12.23,-6.02 | <0.001 | - | - | R |
|  | Sample source |  |  |  |  |  |  |  |
|  | Brain | 1 | -1.73 | -3.09,-0.37 | 0.013 | - | - | R |
|  | Serum | 7 | -4.61 | -6.41,-2.81 | **<0.001** | 88.8 | <0.001 | R |
|  | BALF | 2 | -4.62 | -6.25,-2.98 | **<0.001** | 0.0 | 0.935 | F |
|  | kidney | 1 | -3.11 | -5.80,-0.43 | 0.023 | - | - | R |
|  | Animal species |  |  |  |  |  |  |  |
|  | Mice | 3 | -4.21 | -5.61,-2.81 | **<0.001** | 0.0 | 0.642 | F |
|  | Rats | 8 | -4.15 | -5.71,-2.59 | **<0.001** | 87.5 | <0.001 | R |
| CRP | Nanomaterial types |  |  |  |  |  |  |  |
|  | TiO_2_NPs | 3 | -2.09 | -2.73,-1.45 | **<0.001** | 0.0 | 0.936 | F |
|  | ZnONPs | 4 | -9.21 | -13.50,-4.92 | **<0.001** | 86.8 | <0.001 | R |
|  | Flavonoid subclasses |  |  |  |  |  |  |  |
|  | Flavonols | 6 | -5.49 | -7.87,-3.11 | **<0.001** | 91.5 | <0.001 | R |
|  | (Quercetin) | 6 | -5.49 | -7.87,-3.11 | **<0.001** | 91.5 | <0.001 | R |
|  | Flavanones | 1 | -4.35 | -6.57,-2.13 | <0.001 | - | - | R |
|  | (Hesperidin) | 1 | -4.35 | -6.57,-2.13 | <0.001 | - | - | R |
|  | Flavonoid dosage |  |  |  |  |  |  |  |
|  | ≤100 mg/kg | 1 | -4.35 | -6.57,-2.13 | <0.001 | - | - | R |
|  | > 100 mg/kg | 6 | -5.49 | -7.87,-3.11 | **<0.001** | 91.5 | <0.001 | R |
|  | Intervention duration |  |  |  |  |  |  |  |
|  | ≤ 2 weeks | 1 | -4.35 | -6.57,-2.13 | <0.001 | - | - | R |
|  | ≤ 4 weeks | 6 | -5.49 | -7.87,-3.11 | **<0.001** | 91.5 | <0.001 | R |
|  | Sample source |  |  |  |  |  |  |  |
|  | Brain | 1 | -4.35 | -6.57,-2.13 | <0.001 | - | - | R |
|  | Serum | 6 | -5.49 | -7.87,-3.11 | **<0.001** | 91.5 | <0.001 | R |
| IgG | Nanomaterial types |  |  |  |  |  |  |  |
|  | TiO_2_NPs | 3 | -6.71 | -10.64,-2.77 | **0.001** | 88.3 | <0.001 | R |
|  | ZnONPs | 3 | -8.14 | -11.85,-4.42 | **<0.001** | 81.5 | 0.005 | R |
| VEGF | Nanomaterial types |  |  |  |  |  |  |  |
|  | TiO_2_NPs | 3 | -8.34 | -12.74,-3.94 | **<0.001** | 86.1 | 0.001 | R |
|  | ZnONPs | 2 | -9.24 | -16.08,-2.39 | **0.008** | 87.6 | 0.005 | R |
| MPO | Flavonoid dosage |  |  |  |  |  |  |  |
|  | ≤50 mg/kg | 5 | -12.57 | -17.27,-7.86 | **<0.001** | 86.7 | <0.001 | R |
|  | ≤100 mg/kg | 5 | -15.14 | -21.10,-9.19 | **<0.001** | 89.1 | <0.001 | R |
|  | Sample source |  |  |  |  |  |  |  |
|  | Brain | 6 | -16.67 | -21.78,-15.57 | **<0.001** | 32.3 | 0.194 | F |
|  | Liver | 2 | -7.41 | -9.25,-5.56 | **<0.001** | 1.2 | 0.314 | F |
|  | Kidney | 2 | -7.17 | -8.94,-5.40 | **<0.001** | 0.0 | 0.902 | F |

TiO_2_NPs, titanium dioxide nanoparticles; ZnONPs, zinc oxide nanoparticles; CNTs, carbon nanotubes; SiONPs, silica dioxide nanoparticles; MSNPs, mesoporous silica nanoparticles; TNF, tumor necrosis factor; IL, interleukin; CRP, C-reactive protein; CRP, C-reactive protein; IgG, immunoglobin G; MPO, myeloperoxidase; BALF, bronchoalveolar fluid; SMD, standardized mean difference; CI, confidence interval; F, fixed-effects; R, random-effects; P_H_-value, significance for heterogeneity; P_E_-value, significance for treatment effects. Bold indicated the outcomes significantly changed by flavonoids (analysis with at least two datasets).
