# Supplementary material for: Effects of Flavonoid Supplementation on Nanomaterial-Induced Toxicity: A Meta-Analysis of Preclinical Animal Studies
Source: Front Nutr. 2022 Jun 14;9:929343. doi: 10.3389/fnut.2022.929343 (PMC9237539; doi:10.3389/fnut.2022.929343)
Supplement: Supplementary file 5 [file Table_4.DOCX]

**Supplementary table 4 Subgroup results for apoptosis indicators**

|  | Studies | No. | SMD | 95%CI | P_E_-value | I^2^ | P_H_-value | Model |
| --- | --- | --- | --- | --- | --- | --- | --- | --- |
| FAS | Nanomaterial types |  |  |  |  |  |  |  |
|  | TiO_2_NPs | 6 | -11.24 | -14.77,-7.70 | **<0.001** | 64.4 | 0.015 | R |
|  | AgNPs | 1 | -11.69 | -15.23,-8.14 | <0.001 | - | - | R |
|  | Intervention duration |  |  |  |  |  |  |  |
|  | ≤2 weeks | 4 | -11.37 | -16.60,-6.14 | **<0.001** | 75.6 | 0.006 | R |
|  | ≤4 weeks | 2 | -12.01 | -15.82,-8.20 | **<0.001** | 0.0 | 0.753 | F |
|  | > 4 weeks | 1 | -11.69 | -15.23,-8.14 | <0.001 | - | - | R |
|  | Flavonoid route |  |  |  |  |  |  |  |
|  | Orally | 1 | -11.69 | -15.23,-8.14 | <0.001 | - | - | R |
|  | Intragastrically | 6 | -11.24 | -14.77,-7.70 | **<0.001** | 64.4 | 0.015 | R |
|  | Sample source |  |  |  |  |  |  |  |
|  | Prostate | 3 | -9.43 | -14.20,-4.66 | **<0.001** | 38.6 | 0.196 | F |
|  | Testis | 3 | -7.06 | -9.62,-4.50 | **<0.001** | 58.7 | 0.064 | R |
| Caspase 3 | Nanomaterial types |  |  |  |  |  |  |  |
|  | TiO_2_NPs | 6 | -9.71 | -11.52,-7.90 | **<0.001** | 0.0 | 0.918 | F |
|  | AgNPs | 1 | -15.29 | -19.88,-10.70 | <0.001 | - | - | F |
|  | Intervention duration |  |  |  |  |  |  |  |
|  | ≤ 2 weeks | 4 | -9.40 | -11.55,-7.26 | **<0.001** | 0.0 | 0.857 | F |
|  | ≤ 4 weeks | 2 | -10.46 | -13.82,-7.11 | **<0.001** | 0.0 | 0.520 | F |
|  | > 4 weeks | 1 | -15.29 | -19.88,-10.70 | <0.001 | - | - | F |
|  | Flavonoid route |  |  |  |  |  |  |  |
|  | Orally | 1 | -15.29 | -19.88,-10.70 | <0.001 | - | - | F |
|  | Intragastrically | 6 | -9.71 | -11.52,-7.90 | **<0.001** | 0.0 | 0.918 | F |
|  | Sample source |  |  |  |  |  |  |  |
|  | Prostate | 3 | -10.28 | -12.97,-7.59 | **<0.001** | 0.0 | 0.804 | F |
|  | Testis | 3 | -10.58 | -12.74,-8.42 | **<0.001** | 49.2 | 0.116 | F |
| Bax | Nanomaterial types |  |  |  |  |  |  |  |
|  | TiO_2_NPs | 9 | -10.16 | -11.57,-8.75 | **<0.001** | 49.4 | 0.045 | F |
|  | AgNPs | 2 | -7.02 | -8.67,-5.37 | <0.001 | 0.0 | 0.461 | F |
|  | SiO_2_NPs | 1 | -9.36 | -16.04,-2.68 | 0.006 | - | - | F |
|  | Flavonoid subclasses |  |  |  |  |  |  |  |
|  | Flavonols | 11 | -10.07 | -11.92,-8.22 | **<0.001** | 59.0 | 0.007 | R |
|  | (Quercetin) | 1 | -7.82 | -10.53,-5.12 | **<0.001** | - | - | R |
|  | (Morin) | 8 | -10.67 | -13.10,-8.24 | **<0.001** | 58.4 | 0.018 | R |
|  | (Morin + rutin) | 2 | -10.52 | -17.56,-3.47 | **0.003** | 85.3 | <0.001 | R |
|  | Flavones | 1 | -9.36 | -16.04,-2.68 | 0.006 | - | - | R |
|  | (Apigenin) | 1 | -9.36 | -16.04,-2.68 | 0.006 | - | - | R |
|  | Flavonoid dosage |  |  |  |  |  |  |  |
|  | ≤ 50 mg/kg | 10 | -9.94 | -11.83,-8.05 | **<0.001** | 48.8 | 0.041 | R |
|  | ≤100 mg/kg | 1 | -7.21 | -9.72,-4.70 | **<0.001** | - | - | R |
|  | > 100 mg/kg | 1 | -14.42 | -19.21,-9.63 | **<0.001** | - | - | R |
|  | Intervention duration |  |  |  |  |  |  |  |
|  | ≤ 2 weeks | 4 | -10.88 | -13.38,-8.38 | **<0.001** | 0.0 | 0.399 | F |
|  | ≤ 4 weeks | 3 | -12.95 | -16.93,-8.98 | **<0.001** | 7.4 | 0.339 | F |
|  | > 4 weeks | 5 | -8.50 | -10.56,-6.43 | **<0.001** | 70.5 | 0.017 | R |
|  | Sample source |  |  |  |  |  |  |  |
|  | Prostate | 3 | -12.71 | -17.98,-7.44 | **<0.001** | 58.4 | 0.091 | R |
|  | Testis | 7 | -9.88 | -12.19,-7.58 | **<0.001** | 62.9 | 0.013 | R |
|  | Brain | 1 | -7.82 | -10.53,-5.12 | <0.001 | - | - | R |
|  | Lung | 1 | -9.36 | -16.04,-2.68 | 0.006 | - | - | R |
|  | Flavonoid route |  |  |  |  |  |  |  |
|  | Orally | 3 | -7.16 | -8.76,-5.55 | <0.001 | 0.0 | 0.610 | F |
|  | Intragastrically | 9 | -10.16 | -11.57,-8.75 | **<0.001** | 49.4 | 0.045 | F |
|  | Animal species |  |  |  |  |  |  |  |
|  | Mice | 1 | -9.36 | -16.04,-2.68 | 0.006 | - | - | R |
|  | Rats | 11 | -10.07 | -11.92,-8.22 | **0.006** | 59.0 | 0.007 | R |
| Bcl2 | Nanomaterial types |  |  |  |  |  |  |  |
|  | TiO_2_NPs | 6 | 10.45 | 6.62,14.27 | **<0.001** | 76.7 | 0.001 | R |
|  | AgNPs | 2 | 10.85 | -1.06,22.76 | 0.074 | 93.8 | <0.001 | R |
|  | SiO_2_NPs | 1 | 1.02 | -0.73,2.76 | 0.255 | - | - | R |
|  | Flavonoid subclasses |  |  |  |  |  |  |  |
|  | Flavonols | 8 | 10.32 | 7.08,13.56 | **<0.001** | 82.3 | <0.001 | R |
|  | (Quercetin) | 1 | 17.25 | 11.55,22.96 | <0.001 | - | - | R |
|  | (Morin) | 7 | 9.11 | 6.14,12.08 | **<0.001** | 77.4 | <0.001 | R |
|  | Flavones | 1 | 1.02 | -0.73,2.76 | 0.255 | - | - | R |
|  | (Apigenin) | 1 | 1.02 | -0.73,2.76 | 0.255 | - | - | R |
|  | Intervention duration |  |  |  |  |  |  |  |
|  | ≤ 2 weeks | 4 | 8.52 | 4.63,12.40 | **<0.001** | 74.3 | 0.009 | R |
|  | ≤ 4 weeks | 3 | 9.83 | -0.91,20.57 | 0.073 | 93.2 | <0.001 | R |
|  | > 4 weeks | 2 | 10.85 | -1.06,22.76 | 0.074 | 93.8 | <0.001 | R |
|  | Sample source |  |  |  |  |  |  |  |
|  | Prostate | 3 | 11.73 | 3.27,20.18 | **0.007** | 86.7 | 0.001 | R |
|  | Testis | 4 | 8.21 | 4.74,11.67 | **<0.001** | 73.5 | 0.010 | R |
|  | Lung | 1 | 1.02 | -0.73,2.76 | 0.255 | - | - | R |
|  | Brain | 1 | 17.25 | 11.55,22.96 | <0.001 | - | - | R |
|  | Flavonoid route |  |  |  |  |  |  |  |
|  | Orally | 3 | 6.90 | 1.32,12.48 | 0.015 | 94.0 | <0.001 | R |
|  | Intragastrically | 6 | 10.45 | 6.62,14.27 | **<0.001** | 76.7 | 0.001 | R |
|  | Animal species |  |  |  |  |  |  |  |
|  | Mice | 1 | 1.02 | -0.73,2.76 | 0.255 | - | - | R |
|  | Rats | 8 | 10.32 | 7.08,13.56 | **<0.001** | 82.3 | <0.001 | R |

TiO_2_NPs, titanium dioxide nanoparticles; AgNPs, silver nanoparticles; SMD, standardized mean difference; CI, confidence interval; F, fixed-effects; R, random-effects; P_H_-value, significance for heterogeneity; P_E_-value, significance for treatment effects. Bold indicated the outcomes significantly changed by flavonoids (analysis with at least two datasets).
