# Supplementary material for: Effects of Flavonoid Supplementation on Nanomaterial-Induced Toxicity: A Meta-Analysis of Preclinical Animal Studies
Source: Front Nutr. 2022 Jun 14;9:929343. doi: 10.3389/fnut.2022.929343 (PMC9237539; doi:10.3389/fnut.2022.929343)
Supplement: Supplementary file 6 [file Table_5.DOCX]

**Supplementary table 5 Subgroup results for body weight**

| Studies | No. | SMD | 95%CI | P_E_-value | I^2^ | P_H_-value | Model |
| --- | --- | --- | --- | --- | --- | --- | --- |
| Nanomaterial types |  |  |  |  |  |  |  |
| TiO_2_NPs | 5 | 1.68 | 0.54,2.82 | **0.004** | 71.7 | 0.007 | R |
| ZnONPs | 3 | 0.36 | -0.82,1.54 | 0.551 | 77.7 | 0.011 | R |
| Intervention duration |  |  |  |  |  |  |  |
| ≤ 2 weeks | 3 | 1.47 | 0.76,2.18 | **<0.001** | 0.0 | 0.930 | F |
| ≤ 4 weeks | 4 | 1.29 | -0.40,2.98 | 0.134 | 87.5 | <0.001 | R |
| > 4 weeks | 1 | 0.50 | -0.49,1.50 | 0.323 | - | - | R |
| Flavonoid dosage |  |  |  |  |  |  |  |
| ≤ 50 mg/kg | 3 | 2.68 | 0.45,4.92 | **0.019** | 80.5 | 0.006 | R |
| ≤100 mg/kg | 3 | 1.10 | 0.44,1.75 | **0.001** | 9.4 | 0.332 | F |
| > 100 mg/kg | 2 | -0.17 | -1.14,0.81 | 0.739 | 57.9 | 0.123 | F |
| Flavonoid subclasses |  |  |  |  |  |  |  |
| Flavonols | 7 | 1.07 | 0.11,2.03 | **0.028** | 78.3 | <0.001 | R |
| Quercetin | 4 | 0.35 | -0.46,1.15 | 0.397 | 63.6 | 0.041 | R |
| Morin | 3 | 2.68 | 0.45,4.92 | **0.019** | 80.5 | 0.006 | R |
| Flavanones | 1 | 1.54 | 0.41,2.68 | 0.008 | - | - | R |
| Naringenin | 1 | 1.54 | 0.41,2.68 | 0.008 | - | - | R |
| Animal species |  |  |  |  |  |  |  |
| Mice | 1 | 0.50 | -0.49,1.50 | 0.323 | - | - | R |
| Rats | 7 | 1.26 | 0.25,2.27 | **0.014** | 79.7 | <0.001 | R |
| Flavonoid route |  |  |  |  |  |  |  |
| Orally | 4 | 0.60 | -0.41,1.61 | 0.242 | 75.5 | 0.007 | R |
| Intragastrically | 4 | 1.92 | 0.35,3.49 | **0.017** | 78.7 | 0.003 | R |

TiO_2_NPs, titanium dioxide nanoparticles; ZnONPs, zinc oxide nanoparticles; SMD, standardized mean difference; CI, confidence interval; F, fixed-effects; R, random-effects; P_H_-value, significance for heterogeneity; P_E_-value, significance for treatment effects. Bold indicated the outcomes significantly changed by flavonoids (analysis with at least two datasets).
