# Supplementary material for: Effects of Flavonoid Supplementation on Nanomaterial-Induced Toxicity: A Meta-Analysis of Preclinical Animal Studies
Source: Front Nutr. 2022 Jun 14;9:929343. doi: 10.3389/fnut.2022.929343 (PMC9237539; doi:10.3389/fnut.2022.929343)
Supplement: Supplementary file 7 [file Table_6.DOCX]

**Supplementary table 6 Subgroup results for liver function indicators**

|  | Studies | No. | SMD | 95%CI | P_E_-value | I^2^ | P_H_-value | Model |
| --- | --- | --- | --- | --- | --- | --- | --- | --- |
| ALT | Nanomaterial types |  |  |  |  |  |  |  |
|  | TiO_2_NPs | 5 | -0.95 | -3.16,1.27 | 0.402 | 90.7 | <0.001 | R |
|  | ZnONPs | 1 | -4.80 | -6.83,-2.77 | <0.001 | - | - | R |
|  | AgNPs | 1 | -38.10 | -49.39,-26.82 | <0.001 | - | - | R |
|  | CNTs | 2 | -13.58 | -16.78,-10.39 | **<0.001** | 0.0 | 0.837 | F |
|  | GNPs | 1 | -9.32 | -14.05,-4.59 | <0.001 | - | - | R |
|  | CuONPs | 1 | -58.87 | -78.12,-39.62 | <0.001 | - | - | R |
|  | NiONPs | 3 | -2.58 | -8.15,2.99 | 0.364 | 94.9 | <0.001 | R |
|  | Flavonoid subclasses |  |  |  |  |  |  |  |
|  | Flavonols | 8 | -5.76 | -9.14,-2.38 | **0.001** | 94.8 | <0.001 | R |
|  | (Quercetin) | 7 | -3.25 | -6.17,-0.33 | **0.029** | 93.5 | <0.001 | R |
|  | (Morin) | 1 | -38.10 | -49.39,-26.82 | <0.001 | - | - | R |
|  | Flavanones | 3 | -10.42 | -17.17,-3.68 | **0.002** | 90.3 | <0.001 | R |
|  | (Kolaviron) | 2 | -13.58 | -16.78,-10.39 | **<0.001** | 0.0 | 0.837 | F |
|  | (Naringenin) | 1 | -4.80 | -6.83,-2.77 | 0.001 | - | - | R |
|  | Flavones | 3 | -2.58 | -8.15,2.99 | 0.364 | 94.9 | <0.001 | R |
|  | (Apigenin) | 3 | -2.58 | -8.15,2.99 | 0.364 | 94.9 | <0.001 | R |
|  | Flavonoid dosage |  |  |  |  |  |  |  |
|  | ≤50 mg/kg | 9 | -3.36 | -5.92,-0.79 | **0.010** | 93.8 | <0.001 | R |
|  | ≤100 mg/kg | 2 | -9.12 | -18.06,-0.18 | **0.046** | 92.0 | <0.001 | R |
|  | > 100 mg/kg | 3 | -18.48 | -30.42,-6.53 | **0.002** | 92.6 | <0.001 | R |
|  | Intervention duration |  |  |  |  |  |  |  |
|  | ≤2 weeks | 6 | -2.06 | -4.78,0.67 | 0.139 | 92.2 | <0.001 | R |
|  | ≤4 weeks | 7 | -8.76 | -13.48,-4.03 | **<0.001** | 95.7 | <0.001 | R |
|  | > 4 weeks | 1 | -38.10 | -49.39,-26.82 | <0.001 | - | - | R |
|  | Flavonoid route |  |  |  |  |  |  |  |
|  | Orally | 6 | -2.03 | -4.79,0.74 | **<0.001** | 95.2 | <0.001 | R |
|  | Intraperitoneally | 8 | -8.91 | -13.36,-4.45 | 0.151 | 91.9 | <0.001 | R |
| AST | Nanomaterial types |  |  |  |  |  |  |  |
|  | TiO_2_NPs | 4 | -1.52 | -2.26,-0.78 | **<0.001** | 0.0 | 0.455 | F |
|  | ZnONPs | 1 | -2.39 | -3.71,-1.07 | <0.001 | - | - | R |
|  | AgNPs | 1 | -50.67 | -65.67,-35.68 | <0.001 | - | - | R |
|  | CuONPs | 1 | -109.07 | -144.71,-73.43 | <0.001 | - | - | R |
|  | CNTs | 2 | -13.58 | -17.08,-10.09 | **<0.001** | 14.1 | 0.281 | F |
|  | NiONPs | 3 | -3.04 | -6.13,0.05 | 0.054 | 88.1 | <0.001 | R |
|  | Flavonoid subclasses |  |  |  |  |  |  |  |
|  | Flavonols | 6 | -5.10 | -8.76,-1.45 | **0.006** | 93.6 | <0.001 | R |
|  | (Quercetin) | 5 | -2.23 | -4.83,0.37 | 0.092 | 89.4 | <0.001 | R |
|  | (Morin) | 1 | -50.67 | -65.67,-35.68 | <0.001 | - | - | R |
|  | Flavanones | 3 | -9.86 | -18.79,-0.94 | **0.030** | 95.1 | <0.001 | R |
|  | (Kolaviron) | 2 | -13.58 | -17.08,-10.09 | **<0.001** | 14.1 | 0.281 | F |
|  | (Naringenin) | 1 | -2.39 | -3.71,-1.07 | <0.001 | - | - | R |
|  | Flavones | 3 | -3.04 | -6.13,0.05 | 0.054 | 88.1 | <0.001 | R |
|  | (Apigenin) | 3 | -3.04 | -6.13,0.05 | 0.054 | 88.1 | <0.001 | R |
|  | Flavonoid dosage |  |  |  |  |  |  |  |
|  | ≤ 50 mg/kg | 9 | -3.96 | -6.02,-1.90 | **<0.001** | 90.7 | <0.001 | R |
|  | ≤100 mg/kg | 2 | -8.84 | -21.97,4.29 | 0.187 | 95.8 | <0.001 | R |
|  | > 100 mg/kg | 1 | -109.07 | -144.71,-73.43 | <0.001 | - | - | R |
|  | Intervention duration |  |  |  |  |  |  |  |
|  | ≤ 2 weeks | 5 | -1.87 | -2.95,-0.79 | **0.001** | 63.3 | 0.028 | R |
|  | ≤ 4 weeks | 6 | -8.70 | -13.57,-3.84 | **<0.001** | 94.6 | <0.001 | R |
|  | > 4 weeks | 1 | -50.67 | -65.67,-35.68 | <0.001 | - | - | R |
|  | Flavonoid route |  |  |  |  |  |  |  |
|  | Orally | 7 | -8.15 | -11.83,-4.47 | **<0.001** | 94.3 | <0.001 | R |
|  | Intraperitoneally | 5 | -2.23 | -4.83,0.37 | 0.092 | 89.4 | <0.001 | R |
| ALP | Nanomaterial types |  |  |  |  |  |  |  |
|  | GNPs | 1 | -4.53 | -7.07,-1.99 | <0.001 | - | - | R |
|  | AgNPs | 1 | -46.07 | -59.71,-32.44 | <0.001 | - | - | R |
|  | CNTs | 2 | -6.85 | -14.70,1.01 | 0.088 | 93.7 | <0.001 | R |
|  | Flavonoid subclasses |  |  |  |  |  |  |  |
|  | Flavonols | 2 | -24.74 | -65.44,15.96 | 0.233 | 97.1 | <0.001 | R |
|  | (Quercetin) | 1 | -4.53 | -7.07,-1.99 | <0.001 | - | - | R |
|  | (Morin) | 1 | -46.07 | -59.71,-32.44 | <0.001 | - | - | R |
|  | Flavanones | 2 | -6.85 | -14.70,1.01 | 0.088 | 93.7 | <0.001 | R |
|  | (Kolaviron) | 2 | -6.85 | -14.70,1.01 | 0.088 | 93.7 | <0.001 | R |
|  | Flavonoid dosage |  |  |  |  |  |  |  |
|  | ≤ 50 mg/kg | 2 | -27.92 | -62.22,6.37 | **0.111** | 95.8 | <0.001 | R |
|  | ≤100 mg/kg | 1 | -3.03 | -4.35,-1.71 | <0.001 | - | - | R |
|  | > 100 mg/kg | 1 | -4.53 | -7.07,-1.99 | <0.001 | - | - | R |
|  | Intervention duration |  |  |  |  |  |  |  |
|  | ≤ 2 weeks | 1 | -4.53 | -7.07,-1.99 | <0.001 | - | - | R |
|  | ≤ 4 weeks | 2 | -6.85 | -14.70,1.01 | 0.088 | 93.7 | <0.001 | R |
|  | > 4 weeks | 1 | -46.07 | -59.71,-32.44 | <0.001 | - | - | R |
|  | Flavonoid route |  |  |  |  |  |  |  |
|  | Orally | 3 | -16.85 | -28.63,-5.07 | **0.005** | 96.2 | <0.001 | R |
|  | Intraperitoneally | 1 | -4.53 | -7.07,-1.99 | <0.001 | - | - | R |
| Albumin | Nanomaterial types |  |  |  |  |  |  |  |
|  | CuONPs | 1 | 18.68 | 12.51,24.84 | <0.001 | - | - | R |
|  | NiONPs | 3 | 5.14 | 0.39,9.90 | **0.034** | 89.5 | <0.001 | R |
|  | Flavonoid subclasses |  |  |  |  |  |  |  |
|  | Flavonols | 1 | 18.68 | 12.51,24.84 | <0.001 | - | - | R |
|  | (Quercetin) | 1 | 18.68 | 12.51,24.84 | <0.001 | - | - | R |
|  | Flavones | 3 | 5.14 | 0.39,9.90 | **0.034** | 89.5 | <0.001 | R |
|  | (Apigenin) | 3 | 5.14 | 0.39,9.90 | **0.034** | 89.5 | <0.001 | R |
|  | Flavonoid dosage |  |  |  |  |  |  |  |
|  | ≤ 50 mg/kg | 3 | 5.14 | 0.39,9.90 | **0.034** | - | - | R |
|  | > 100 mg/kg | 1 | 18.68 | 12.51,24.84 | <0.001 | 89.5 | <0.001 | R |
|  | Intervention duration |  |  |  |  |  |  |  |
|  | ≤ 2 weeks | 2 | 3.89 | -1.60,9.38 | 0.165 | 90.1 | 0.001 | R |
|  | ≤ 4 weeks | 2 | 12.99 | 2.45,23.53 | **0.016** | 88.5 | 0.003 | R |
|  | Flavonoid route |  |  |  |  |  |  |  |
|  | Orally | 3 | 5.14 | 0.39,9.90 | **0.034** | 89.5 | <0.001 | R |
|  | Intraperitoneally | 1 | 18.68 | 12.51,24.84 | <0.001 | - | - | R |

TiO_2_NPs, titanium dioxide nanoparticles; CuONPs, copper oxide nanoparticles; ZnONPs, zinc oxide nanoparticles; GNPs, gold nanoparticles; NiONPs, nickel oxide nanoparticles; AgNPs, silver nanoparticles; CNTs, carbon nanotubes; ALT, alanine aminotransferase; AST, aspartate aminotransferase; ALP, alkaline phosphatase; SMD, standardized mean difference; CI, confidence interval; F, fixed-effects; R, random-effects; P_H_-value, significance for heterogeneity; P_E_-value, significance for treatment effects. Bold indicated the outcomes significantly changed by flavonoids (analysis with at least two datasets).
