# Supplementary material for: Effects of Flavonoid Supplementation on Nanomaterial-Induced Toxicity: A Meta-Analysis of Preclinical Animal Studies
Source: Front Nutr. 2022 Jun 14;9:929343. doi: 10.3389/fnut.2022.929343 (PMC9237539; doi:10.3389/fnut.2022.929343)
Supplement: Supplementary file 8 [file Table_7.DOCX]

**Supplementary table 7 Subgroup results for renal function indicators**

|  | Studies | No. | SMD | 95%CI | P_E_-value | I^2^ | P_H_-value | Model |
| --- | --- | --- | --- | --- | --- | --- | --- | --- |
| Urea | Nanomaterial types |  |  |  |  |  |  |  |
|  | TiO_2_NPs | 2 | -4.06 | -5.70,-2.43 | **<0.001** | 50.4 | 0.156 | R |
|  | ZnONPs | 2 | -0.80 | -1.45,-0.15 | **0.015** | 0.0 | 0.519 | R |
|  | CNTs | 2 | -6.07 | -7.61,-4.53 | **<0.001** | 0.0 | 0.325 | R |
|  | NiONPs | 3 | -4.16 | -7.82,-0.50 | **0.026** | 88.9 | <0.001 | R |
|  | Flavonoid subclasses |  |  |  |  |  |  |  |
|  | Flavonols | 4 | -2.34 | -4.07,-0.62 | **0.008** | 88.1 | <0.001 | R |
|  | (Quercetin) | 4 | -2.34 | -4.07,-0.62 | **0.008** | 88.1 | <0.001 | R |
|  | Flavanones | 2 | -10.39 | -12.93,-7.86 | **<0.001** | 0.0 | 0.325 | R |
|  | (Kolaviron) | 2 | -10.39 | -12.93,-7.86 | **<0.001** | 0.0 | 0.325 | R |
|  | Flavones | 3 | -4.16 | -7.82,-0.50 | **0.026** | 88.9 | <0.001 | R |
|  | (Apigenin) | 3 | -4.16 | -7.82,-0.50 | **0.026** | 88.9 | <0.001 | R |
|  | Flavonoid dosage |  |  |  |  |  |  |  |
|  | ≤ 50 mg/kg | 4 | -4.49 | -7.57,-1.42 | **0.004** | 89.3 | <0.001 | R |
|  | ≤ 100 mg/kg | 1 | -7.03 | -9.49,-4.57 | <0.001 | - | - | R |
|  | > 100 mg/kg | 4 | -2.34 | -4.07,-0.62 | **0.008** | 88.1 | <0.001 | R |
|  | Intervention duration |  |  |  |  |  |  |  |
|  | ≤2 weeks | 2 | -1.80 | -3.76,0.15 | 0.071 | 72.0 | 0.059 | R |
|  | ≤4 weeks | 7 | -4.24 | -6.23,-2.25 | **<0.001** | 91.2 | <0.001 | R |
| Blood urea nitrogen | Nanomaterial types |  |  |  |  |  |  |  |
|  | TiO_2_NPs | 1 | -6.93 | -9.67,-4.18 | <0.001 | - | - | R |
|  | AgNPs | 1 | -26.31 | -34.12,-18.49 | <0.001 | - | - | R |
|  | GNPs | 1 | -4.97 | -7.42,-2.52 | <0.001 | - | - | R |
|  | NiONPs | 3 | -2.56 | -5.11,-0.01 | **0.049** | 85.7 | <0.001 | R |
|  | MSNPs | 1 | -3.53 | -6.45,-0.61 | 0.018 | - | - | R |
|  | Flavonoid subclasses |  |  |  |  |  |  |  |
|  | Flavonols | 3 | -11.40 | -18.75,-4.04 | **0.002** | 92.3 | <0.001 | R |
|  | (Quercetin) | 2 | -5.85 | -7.76,-3.94 | **<0.001** | 7.8 | 0.298 | F |
|  | (Morin) | 1 | -26.31 | -34.12,-18.49 | <0.001 | - | - | F |
|  | Flavones | 4 | -2.73 | -4.90,-0.55 | **0.014** | 81.2 | 0.001 | R |
|  | (Apigenin) | 4 | -2.73 | -4.90,-0.55 | **0.014** | 81.2 | 0.001 | R |
|  | Flavonoid dosage |  |  |  |  |  |  |  |
|  | ≤ 50 mg/kg | 5 | -5.76 | -9.35,-2.16 | **0.002** | 92.6 | <0.001 | R |
|  | ≤100 mg/kg | 2 | -5.85 | -7.76,-3.94 | **<0.001** | 7.8 | 0.298 | R |
|  | Intervention duration |  |  |  |  |  |  |  |
|  | ≤ 2 weeks | 4 | -2.28 | -4.03,-0.54 | **0.010** | 74.6 | 0.008 | R |
|  | ≤ 4 weeks | 2 | -7.25 | -9.44,-5.07 | **<0.001** | 0.0 | 0.700 | F |
|  | > 4 weeks | 1 | -26.31 | -34.12,-18.49 | <0.001 | - | - | R |
|  | Flavonoid route |  |  |  |  |  |  |  |
|  | Orally | 5 | -6.70 | -10.59,-2.80 | **0.001** | 93.9 | <0.001 | R |
|  | Intraperitoneally | 2 | -4.37 | -6.25,-2.50 | <0.001 | 0.0 | 0.460 | F |
|  | Animal species |  |  |  |  |  |  |  |
|  | Mice | 1 | -3.53 | -6.45,-0.61 | 0.018 | - | - | R |
|  | Rats | 6 | -6.26 | -9.59,-2.93 | **<0.001** | 92.9 | <0.001 | R |
| Creatinine | Nanomaterial types |  |  |  |  |  |  |  |
|  | TiO_2_NPs | 3 | -8.35 | -10.07,-6.62 | **<0.001** | 0.0 | 0.597 | R |
|  | NiONPs | 3 | -2.25 | -4.13,-0.38 | **0.019** | 76.2 | 0.015 | R |
|  | ZnONPs | 2 | -1.13 | -1.89,-0.37 | **0.004** | 20.5 | 0.262 | R |
|  | GNPs | 1 | -7.00 | -10.27,-3.73 | <0.001 | - | - | R |
|  | AgNPs | 1 | -56.29 | -72.94,-39.64 | **<0.001** | - | - | R |
|  | CNTs | 2 | -7.06 | -8.81,-5.31 | **<0.001** | 0.0 | 0.663 | R |
|  | MSNPs | 1 | -2.00 | -4.12,0.12 | 0.064 | - | - | R |
|  | Flavonoid subclasses |  |  |  |  |  |  |  |
|  | Flavonols | 7 | -5.56 | -7.54,-3.58 | **<0.001** | 94.5 | <0.001 | R |
|  | (Quercetin) | 6 | -5.51 | -8.24,-2.78 | **<0.001** | 92.7 | <0.001 | R |
|  | (Morin) | 1 | -56.29 | -72.94,-39.64 | 0.002 | - | - | R |
|  | Flavanones | 2 | -7.06 | -8.81,-5.31 | **<0.001** | 0.0 | 0.663 | R |
|  | (Kolaviron) | 2 | -7.06 | -8.81,-5.31 | **<0.001** | 0.0 | 0.663 | R |
|  | Flavones | 4 | -2.12 | -3.54,-0.70 | **0.003** | 64.6 | 0.037 | R |
|  | (Apigenin) | 4 | -2.12 | -3.54,-0.70 | **0.003** | 64.6 | 0.037 | R |
|  | Flavonoid dosage |  |  |  |  |  |  |  |
|  | ≤ 50 mg/kg | 6 | -4.78 | -7.86,-1.71 | **0.002** | 92.2 | <0.001 | R |
|  | ≤ 100 mg/kg | 3 | -7.90 | -9.70,-6.10 | **<0.001** | 0.0 | 0.453 | R |
|  | > 100 mg/kg | 4 | -4.20 | -7.03,-1.38 | **0.004** | 93.1 | <0.001 | R |
|  | Intervention duration |  |  |  |  |  |  |  |
|  | ≤ 2 weeks | 4 | -2.34 | -4.05,-0.63 | **0.007** | 73.2 | 0.011 | R |
|  | ≤ 4 weeks | 8 | -5.71 | -8.09,-3.33 | **<0.001** | 92.4 | <0.001 | R |
|  | > 4 weeks | 1 | -56.29 | -72.94,-39.64 | <0.001 | - | - | R |
|  | Flavonoid route |  |  |  |  |  |  |  |
|  | Orally | 11 | -5.43 | -7.47,-3.38 | **<0.001** | 92.9 | <0.001 | R |
|  | Intragastrically | 2 | -4.34 | -9.23,0.55 | 0.082 | 84.2 | 0.012 | R |
|  | Animal species |  |  |  |  |  |  |  |
|  | Mice | 12 | -5.56 | -7.54,-3.58 | **<0.001** | 92.6 | <0.001 | R |
|  | Rats | 1 | -2.00 | -4.12,0.12 | 0.064 | - | - | R |
| Uric acid | Nanomaterial types |  |  |  |  |  |  |  |
|  | TiO_2_NPs | 3 | -5.01 | -6.81,-3.21 | **<0.001** | 59.9 | 0.083 | R |
|  | ZnONPs | 2 | -0.17 | -0.79,0.46 | 0.603 | 0.0 | 0.842 | R |
|  | AgNPs | 1 | -12.11 | -15.78,-8.45 | **<0.001** | - | - | R |
|  | GNPs | 1 | -0.73 | -1.90,0.45 | 0.225 | - | - | R |
|  | Flavonoid subclasses |  |  |  |  |  |  |  |
|  | Flavonols | 7 | -3.63 | -5.68,-1.58 | **0.001** | 93.3 | <0.001 | R |
|  | (Quercetin) | 6 | -2.53 | -4.29,-0.77 | **0.005** | 91.1 | <0.001 | R |
|  | (Morin) | 1 | -12.11 | -15.78,-8.45 | <0.001 | - | - | R |
|  | Flavonoid dosage |  |  |  |  |  |  |  |
|  | ≤ 50 mg/kg | 1 | -12.11 | -15.78,-8.45 | **<0.001** | - | - | R |
|  | ≤100 mg/kg | 2 | -2.71 | -6.76,1.35 | 0.190 | 91.5 | 0.001 | R |
|  | > 100 mg/kg | 4 | -2.52 | -4.83,-0.20 | **0.033** | 93.0 | <0.001 | R |
|  | Intervention duration |  |  |  |  |  |  |  |
|  | ≤ 2 weeks | 1 | -0.73 | -1.90,0.45 | 0.225 | - | - | R |
|  | ≤ 4 weeks | 5 | -2.98 | -5.18,-0.78 | **0.008** | 92.8 | <0.001 | R |
|  | > 4 weeks | 1 | -12.11 | -15.78,-8.45 | **<0.001** | - | - | R |
|  | Flavonoid route |  |  |  |  |  |  |  |
|  | Orally | 6 | -4.26 | -6.77,-1.75 | **0.001** | 94.3 | <0.001 | R |
|  | Intraperitoneally | 1 | -0.73 | -1.90,0.48 | 0.225 | - | - | R |

TiO_2_NPs, titanium dioxide nanoparticles; ZnONPs, zinc oxide nanoparticles; GNPs, gold nanoparticles; NiONPs, nickel oxide nanoparticles; AgNPs, silver nanoparticles; CNTs, carbon nanotubes; NSNPs, mesoporous silica nanoparticles; SMD, standardized mean difference; CI, confidence interval; F, fixed-effects; R, random-effects; P_H_-value, significance for heterogeneity; P_E_-value, significance for treatment effects. Bold indicated the outcomes significantly changed by flavonoids (analysis with at least two datasets).
