# Supplementary material for: Effects of Flavonoid Supplementation on Nanomaterial-Induced Toxicity: A Meta-Analysis of Preclinical Animal Studies
Source: Front Nutr. 2022 Jun 14;9:929343. doi: 10.3389/fnut.2022.929343 (PMC9237539; doi:10.3389/fnut.2022.929343)
Supplement: Supplementary file 9 [file Table_8.DOCX]

**Supplementary table 8 Subgroup results for** **testis function indicators**

|  | Studies | No. | SMD | 95%CI | P_E_-value | I^2^ | P_H_-value | Model |
| --- | --- | --- | --- | --- | --- | --- | --- | --- |
| Testosterone | Nanomaterial types |  |  |  |  |  |  |  |
|  | TiO_2_NPs | 7 | 7.72 | 4.14,11.30 | **<0.001** | 91.3 | <0.001 | R |
|  | ZnONPs | 2 | 11.21 | -8.76,31.17 | 0.271 | 96.7 | <0.001 | R |
|  | AgNPs | 1 | 3.45 | 2.16,4.75 | <0.001 | - | - | R |
|  | Flavonoid subclasses |  |  |  |  |  |  |  |
|  | Flavonols | 10 | 6.97 | 4.48,9.46 | **<0.001** | 92.3 | <0.001 | R |
|  | (Quercetin) | 3 | 8.66 | 1.60,15.72 | **0.016** | 95.2 | <0.001 | R |
|  | (Rutin) | 1 | 54.37 | 36.59,72.15 | <0.001 | - | - | R |
|  | (Morin) | 5 | 4.84 | 2.55,7.12 | **<0.001** | 84.6 | <0.001 | R |
|  | (Morin + rutin) | 1 | 8.59 | 5.65,11.53 | <0.001 | - | - | R |
|  | Flavonoid dosage |  |  |  |  |  |  |  |
|  | ≤ 50 mg/kg | 5 | 4.84 | 2.55,7.12 | **<0.001** | 84.6 | <0.001 | R |
|  | ≤100 mg/kg | 4 | 15.10 | 6.55,23.65 | **0.001** | 96.0 | <0.001 | R |
|  | 100 mg/kg | 1 | 8.59 | 5.65,11.53 | <0.001 | - | - | R |
|  | Intervention duration |  |  |  |  |  |  |  |
|  | ≤2 weeks | 2 | 5.94 | 3.91,7.96 | **<0.001** | 0.0 | 0.397 | F |
|  | ≤4 weeks | 1 | 1.54 | 0.22,2.86 | 0.022 | - | - | R |
|  | > 4 weeks | 7 | 8.95 | 5.29,12.62 | **<0.001** | 94.2 | <0.001 | R |
|  | Flavonoid route |  |  |  |  |  |  |  |
|  | Orally | 1 | 3.45 | 2.16,4.75 | <0.001 | - | - | R |
|  | Intraperitoneally | 9 | 7.89 | 4.82,10.96 | **<0.001** | 93.1 | <0.001 | R |
|  | Animal models |  |  |  |  |  |  |  |
|  | Mice | 1 | 6.40 | 3.83,8.96 | <0.001 | - | - | R |
|  | Rats | 9 | 7.09 | 4.40,9.78 | **<0.001** | 92.8 | <0.001 | R |
| FSH | Nanomaterial types |  |  |  |  |  |  |  |
|  | TiO_2_NPs | 6 | 3.26 | -2.54,9.06 | 0.271 | 96.4 | <0.001 | R |
|  | AgNPs | 1 | 2.33 | 1.27,3.38 | <0.001 | - | - | R |
|  | Flavonoid subclasses |  |  |  |  |  |  |  |
|  | Flavonols | 7 | 2.61 | -1.50,6.71 | 0.213 | 95.9 | <0.001 | R |
|  | (Rutin) | 1 | 4.94 | 3.10,6.78 | **<0.001** | - | - | R |
|  | (Morin) | 5 | 0.65 | -4.33,5.63 | 0.799 | 95.9 | <0.001 | R |
|  | (Morin + rutin) | 1 | 9.50 | 6.27,12.72 | **<0.001** | - | - | R |
|  | Flavonoid dosage |  |  |  |  |  |  |  |
|  | ≤ 50 mg/kg | 5 | 0.65 | -4.33,5.63 | 0.799 | 95.9 | <0.001 | R |
|  | ≤100 mg/kg | 1 | 4.94 | 3.10,6.78 | <0.001 | - | - | R |
|  | > 100 mg/kg | 1 | 9.50 | 6.27,12.72 | <0.001 | - | - | R |
|  | Intervention duration |  |  |  |  |  |  |  |
|  | ≤ 2 weeks | 2 | -5.21 | -11.63,1.22 | 0.112 | 89.0 | 0.003 | R |
|  | ≤ 4 weeks | 1 | -5.88 | -8.69,-3.07 | **<0.001** | - | - | R |
|  | > 4 weeks | 4 | 8.75 | 3.95,13.55 | **<0.001** | 93.8 | <0.001 | R |
|  | Flavonoid route |  |  |  |  |  |  |  |
|  | Orally | 1 | 2.33 | 1.27,3.38 | **<0.001** | - | - | R |
|  | Intragastrically | 6 | 3.26 | -2.54,9.06 | 0.271 | 96.4 | <0.001 | R |
| LH | Nanomaterial types |  |  |  |  |  |  |  |
|  | TiO_2_NPs | 6 | 2.24 | -2.72,7.20 | 0.376 | 96.3 | <0.001 | R |
|  | AgNPs | 1 | 8.10 | 5.57,10.62 | **<0.001** | 0.0 | 0.351 | F |
|  | Flavonoid subclasses |  |  |  |  |  |  |  |
|  | Flavonols | 7 | 3.09 | -1.60,7.78 | 0.196 | 96.4 | <0.001 | R |
|  | (Rutin) | 1 | 14.00 | 9.35,18.66 | <0.001 | 0.8 | 0.315 | F |
|  | (Morin) | 5 | 0.59 | -4.67,5.85 | 0.827 | 96.4 | <0.001 | R |
|  | (Morin + rutin) | 1 | 5.73 | 3.66,7.80 | <0.001 | - | - | R |
|  | Flavonoid dosage |  |  |  |  |  |  |  |
|  | ≤ 50 mg/kg | 5 | 0.59 | -4.67,5.85 | 0.827 | 96.4 | <0.001 | R |
|  | ≤100 mg/kg | 1 | 14.00 | 9.35,18.66 | <0.001 | - | - | R |
|  | > 100 mg/kg | 1 | 5.73 | 3.66,7.80 | <0.001 | - | - | R |
|  | Intervention duration |  |  |  |  |  |  |  |
|  | ≤ 2 weeks | 2 | -3.08 | -4.69,-1.46 | **<0.001** | 36.7 | 0.209 | F |
|  | ≤ 4 weeks | 1 | -5.94 | -8.78,-3.10 | <0.001 | - | - | R |
|  | > 4 weeks | 4 | 8.17 | 5.60,10.75 | **<0.001** | 71.5 | 0.015 | R |
|  | Flavonoid route |  |  |  |  |  |  |  |
|  | Orally | 1 | 8.10 | 5.57,10.62 | <0.001 | - | - | R |
|  | Intragastrically | 6 | 2.24 | -2.72,7.20 | 0.376 | 96.3 | <0.001 | R |
| Sperm motility | Nanomaterial types |  |  |  |  |  |  |  |
|  | TiO_2_NPs | 3 | 10.40 | 8.364,12.44 | **<0.001** | 0.0 | 0.452 | F |
|  | ZnONPs | 2 | 5.04 | -2.28,12.37 | 0.177 | 95.2 | <0.001 | R |
|  | AgNPs | 1 | 4.91 | 3.25,6.57 | <0.001 | - | - | R |
|  | Flavonoid subclasses |  |  |  |  |  |  |  |
|  | Flavonols | 6 | 7.64 | 4.06,11.22 | **<0.001** | 93.4 | <0.001 | R |
|  | (Quercetin) | 2 | 5.04 | -2.28,12.37 | 0.177 | 95.2 | <0.001 | R |
|  | (Rutin) | 1 | 8.94 | 5.89,11.99 | 0.001 | - | - | R |
|  | (Morin) | 2 | 8.02 | 1.46,14.59 | **0.017** | 89.6 | 0.002 | R |
|  | (Morin + rutin) | 1 | 11.53 | 7.66,15.40 | <0.001 | - | - | R |
|  | Flavonoid dosage |  |  |  |  |  |  |  |
|  | ≤ 50 mg/kg | 2 | 8.02 | 1.46,14.59 | **0.017** | 89.6 | 0.002 | R |
|  | ≤100 mg/kg | 3 | 6.30 | 0.44,12.17 | **0.035** | 94.8 | <0.001 | R |
|  | > 100 mg/kg | 1 | 11.53 | 7.66,15.40 | <0.001 | - | - | R |
|  | Flavonoid route |  |  |  |  |  |  |  |
|  | Orally | 1 | 4.91 | 3.25,6.57 | <0.001 | - | - | R |
|  | Intragastrically | 5 | 8.34 | 3.25,13.43 | **0.001** | 94.6 | <0.001 | R |
| Sperm count | Nanomaterial types |  |  |  |  |  |  |  |
|  | TiO_2_NPs | 4 | 9.15 | 4.25,14.05 | **<0.001** | 90.8 | <0.001 | R |
|  | ZnONPs | 2 | 1.39 | -2.78,5.55 | 0.514 | 95.8 | <0.001 | R |
|  | AgNPs | 1 | 4.58 | 3.01,6.15 | <0.001 | - | - | R |
|  | Flavonoid subclasses |  |  |  |  |  |  |  |
|  | Flavonols | 7 | 5.95 | 2.93,8.97 | **<0.001** | 94.9 | <0.001 | R |
|  | (Quercetin) | 3 | 2.05 | -1.06,5.15 | 0.197 | 94.1 | <0.001 | R |
|  | (Rutin) | 1 | 7.87 | 5.15,10.58 | <0.001 | - | - | R |
|  | (Morin) | 2 | 8.12 | 0.73,15.51 | **0.031** | 91.4 | 0.001 | R |
|  | (Morin + rutin) | 1 | 14.54 | 9.71,19.37 | <0.001 | - | - | R |
|  | Flavonoid dosage |  |  |  |  |  |  |  |
|  | ≤ 50 mg/kg | 2 | 8.12 | 0.73,15.51 | **0.031** | 91.4 | <0.001 | R |
|  | ≤100 mg/kg | 4 | 3.38 | 0.12,6.64 | **0.042** | 94.7 | <0.001 | R |
|  | >100 mg/kg | 1 | 14.54 | 9.71,19.37 | <0.001 | - | - | R |
|  | Flavonoid route |  |  |  |  |  |  |  |
|  | Orally | 1 | 4.58 | 3.01,6.15 | <0.001 | - | - | R |
|  | Intragastrically | 6 | 6.29 | 2.70,9.88 | **0.001** | 95.4 | <0.001 | R |
|  | Animal models |  |  |  |  |  |  |  |
|  | Mice | 1 | 3.43 | 1.83,5.04 | <0.001 | - | - | R |
|  | Rats | 6 | 6.52 | 2.84,10.19 | **0.001** | 95.7 | <0.001 | R |
| Sperm abnormalities | Nanomaterial types |  |  |  |  |  |  |  |
|  | TiO_2_NPs | 4 | -8.48 | -11.12,-5.83 | **<0.001** | 67.9 | 0.025 | R |
|  | AgNPs | 1 | -5.66 | -7.51,-3.81 | <0.001 | - | - | R |
|  | Flavonoid subclasses |  |  |  |  |  |  |  |
|  | Flavonols | 5 | -7.73 | -9.87,-5.60 | **<0.001** | 68.2 | 0.014 | R |
|  | (Quercetin) | 1 | -6.05 | -8.49,-3.60 | <0.001 | - | - | R |
|  | (Rutin) | 1 | -10.33 | -13.81,-6.84 | <0.001 | - | - | R |
|  | (Morin) | 2 | -8.73 | -15.26,-2.21 | **0.009** | 88.1 | 0.004 | R |
|  | (Morin + rutin) | 1 | -6.78 | -9.17,-4.40 | <0.001 | - | - | R |
|  | Flavonoid dosage |  |  |  |  |  |  |  |
|  | ≤ 50 mg/kg | 2 | -8.73 | -15.26,-2.21 | **0.009** | 88.1 | 0.004 | R |
|  | ≤100 mg/kg | 2 | -8.00 | -12.18,-3.82 | **<0.001** | 74.2 | 0.049 | R |
|  | >100 mg/kg | 1 | -6.78 | -9.17,-4.40 | <0.001 | - | - | R |
|  | Flavonoid route |  |  |  |  |  |  |  |
|  | Orally | 1 | -5.66 | -7.51,-3.81 | <0.001 | - | - | R |
|  | Intragastrically | 4 | -8.48 | -11.12,-5.83 | **<0.001** | 67.9 | 0.025 | R |
|  | Animal models |  |  |  |  |  |  |  |
|  | Mice | 1 | -6.05 | -8.49,-3.60 | <0.001 | - | - | R |
|  | Rats | 4 | -8.33 | -11.10,-5.56 | **<0.001** | 74.6 | 0.008 | R |
| Live sperm | Nanomaterial types |  |  |  |  |  |  |  |
|  | TiO_2_NPs | 3 | 11.30 | 9.10,13.51 | **<0.001** | 0.0 | 0.514 | F |
|  | ZnONPs | 2 | 7.13 | -4.90,19.16 | 0.245 | 96.4 | <0.001 | R |
|  | AgNPs | 1 | -5.66 | -7.51,-3.81 | **<0.001** | - | - | R |
|  | Flavonoid subclasses |  |  |  |  |  |  |  |
|  | Flavonols | 6 | 7.05 | 1.32,12.78 | **0.016** | 96.9 | <0.001 | R |
|  | (Quercetin) | 2 | 7.13 | -4.90,19.16 | 0.245 | 96.4 | <0.001 | R |
|  | (Rutin) | 1 | 9.84 | 6.51,13.17 | <0.001 | - | - | R |
|  | (Morin) | 2 | 3.33 | -14.48,21.14 | 0.714 | 98.4 | <0.001 | R |
|  | (Morin + rutin) | 1 | 12.39 | 8.25,16.52 | <0.001 | - | - | R |
|  | Flavonoid dosage |  |  |  |  |  |  |  |
|  | ≤ 50 mg/kg | 2 | 3.33 | -14.48,21.14 | 0.714 | 98.4 | <0.001 | R |
|  | ≤100 mg/kg | 3 | 7.96 | -0.06,15.98 | 0.052 | 95.9 | <0.001 | R |
|  | >100 mg/kg | 1 | 12.39 | 8.25,16.52 | <0.001 | - | - | R |
|  | Flavonoid route |  |  |  |  |  |  |  |
|  | Orally | 1 | -5.66 | -7.51,-3.81 | <0.001 | - | - | R |
|  | Intragastrically | 5 | 9.73 | 3.32,16.13 | **0.003** | 95.6 | <0.001 | R |
| 17β-HSD | Nanomaterial types |  |  |  |  |  |  |  |
|  | TiO_2_NPs | 3 | 7.05 | 5.61,8.49 | **<0.001** | 36.6 | 0.207 | F |
|  | ZnONPs | 2 | 2.90 | 1.99,3.82 | **<0.001** | 0.0 | 0.606 | F |
|  | Flavonoid subclasses |  |  |  |  |  |  |  |
|  | Flavonols | 5 | 5.35 | 3.21,7.49 | **<0.001** | 84.6 | <0.001 | R |
|  | (Quercetin) | 2 | 2.90 | 1.99,7.80 | **<0.001** | 0.0 | 0.606 | F |
|  | (Rutin) | 1 | 5.74 | 3.67,7.80 | <0.001 | - | - | F |
|  | (Morin) | 1 | 7.97 | 5.22,10.71 | <0.001 | - | - | F |
|  | (Morin + rutin) | 1 | 8.69 | 5.72,11.66 | <0.001 | - | - | F |
|  | Flavonoid dosage |  |  |  |  |  |  |  |
|  | ≤ 50 mg/kg | 1 | 7.97 | 5.22,10.71 | <0.001 | - | - | R |
|  | ≤100 mg/kg | 3 | 3.37 | 2.53,4.20 | **<0.001** | 68.2 | 0.043 | R |
|  | > 100 mg/kg | 1 | 8.69 | 5.72,11.66 | <0.001 | - | - | R |

TiO_2_NPs, titanium dioxide nanoparticles; ZnONPs, zinc oxide nanoparticles; AgNPs, silver nanoparticles; LH, luteinizing hormone; FSH, follicle-stimulating hormone; 17β-HSD, 17β-Hydroxysteroid dehydrogenase type; SMD, standardized mean difference; CI, confidence interval; F, fixed-effects; R, random-effects; P_H_-value, significance for heterogeneity; P_E_-value, significance for treatment effects. Bold indicated the outcomes significantly changed by flavonoids (analysis with at least two datasets).
