# Supplementary material for: Effects of Flavonoid Supplementation on Nanomaterial-Induced Toxicity: A Meta-Analysis of Preclinical Animal Studies
Source: Front Nutr. 2022 Jun 14;9:929343. doi: 10.3389/fnut.2022.929343 (PMC9237539; doi:10.3389/fnut.2022.929343)
Supplement: Supplementary file 10 [file Table_9.DOCX]

**Supplementary table 9 Subgroup results for brain function indicators**

|  | Studies | No. | SMD | 95%CI | P_E_-value | I^2^ | P_H_-value | Model |
| --- | --- | --- | --- | --- | --- | --- | --- | --- |
| Serotonin | Nanomaterial types |  |  |  |  |  |  |  |
|  | AgNPs | 1 | 151.33 | 98.89,203.77 | <0.001 | - | - | F |
|  | IONPs | 3 | 1.70 | 1.02,2.38 | **<0.001** | 0.0 | 0.368 | F |
|  | Flavonoid subclasses |  |  |  |  |  |  |  |
|  | Flavonols | 4 | 2.18 | -0.57,4.93 | 0.120 | 91.0 | <0.001 | R |
|  | (Quercetin) | 3 | 1.70 | 1.02,2.38 | **<0.001** | 0.0 | 0.368 | F |
|  | (Rutin) | 1 | 151.33 | 98.89,203.77 | <0.001 | - | - | F |
|  | Flavonoid dosage |  |  |  |  |  |  |  |
|  | ≤50 mg/kg | 3 | 2.56 | -1.89,7.02 | 0.259 | 93.8 | <0.001 | R |
|  | ≤100 mg/kg | 1 | 2.34 | 1.03,3.65 | **<0.001** | - | - | R |
| AChE | Nanomaterial types |  |  |  |  |  |  |  |
|  | IONP | 3 | -3.88 | -5.46,-2.30 | **<0.001** | 57.5 | 0.095 | R |
|  | CNTs | 6 | 7.67 | 6.55,8.79 | **<0.001** | 3.9 | 0.391 | F |
|  | AgNPs | 1 | 8.51 | 5.59,11.42 | **<0.001** | - | - | R |
|  | Flavonoid subclasses |  |  |  |  |  |  |  |
|  | Flavonols | 4 | -1.05 | -5.63,-3.53 | 0.654 | 95.3 | <0.001 | R |
|  | (Quercetin) | 4 | -1.05 | -5.63,-3.53 | 0.654 | 95.3 | <0.001 | R |
|  | Flavanones | 6 | 7.67 | 6.55,8.79 | **<0.001** | 3.9 | 0.391 | F |
|  | (Kolaviron) | 6 | 7.67 | 6.55,8.79 | **<0.001** | 3.9 | 0.391 | F |
|  | Flavonoid dosage |  |  |  |  |  |  |  |
|  | ≤ 50 mg/kg | 6 | 3.89 | -0.98,8.76 | 0.117 | 96.7 | <0.001 | R |
|  | ≤100 mg/kg | 4 | 5.17 | -2.49,12.82 | 0.186 | 96.9 | <0.001 | R |
|  | Intervention duration |  |  |  |  |  |  |  |
|  | ≤ 4 weeks | 6 | 7.67 | 6.55,8.79 | **<0.001** | 3.9 | 0.391 | F |
|  | > 4 weeks | 4 | -1.05 | -5.63,-3.53 | 0.654 | 95.3 | <0.001 | R |

AgNPs, silver nanoparticles; CNTs, carbon nanotubes; IONPs, iron oxide nanoparticles; AChE, acetylcholinesterase; SMD, standardized mean difference; CI, confidence interval; F, fixed-effects; R, random-effects; P_H_-value, significance for heterogeneity; P_E_-value, significance for treatment effects. Bold indicated the outcomes significantly changed by flavonoids (analysis with at least two datasets).
